# Tutorial for Atomistic and Coarse-Grained Simulations of Membrane-Nanoplastic Interactions Using GROMACS

**DOI:** 10.1021/acs.jpcb.6c01672

**Published:** 2026-07-29

**Authors:** Sasthi C. Mandal, Atanu Acharya

**Affiliations:** † Department of Chemistry, 2029 Syracuse University, Syracuse, New York 13244, United States; ‡ BioInspired Syracuse, Syracuse University, Syracuse, New York 13244, United States

## Abstract

Molecular dynamics (MD) simulations are widely used to investigate membrane properties and their interactions with various plastic particles. In this tutorial, we provide guidelines for both atomistic and coarse-grained MD simulations to investigate interactions between a POPC membrane and a polystyrene (PS) nanoplastic. This tutorial is organized into a few exercises focusing on three systems: (i) PS in water, (ii) POPC membrane in water, and (iii) POPC–PS complex in water, each simulated at atomistic and coarse-grained levels. The aim of this tutorial is to familiarize readers with the process of modeling nanoplastic and membrane systems using CHARMM-GUI and performing MD simulations with GROMACS. In addition, we calculate a few biophysical properties, including the radius of gyration of PS, membrane thickness, area per lipid, order parameters, and diffusion coefficient of POPC lipids, using GROMACS analysis tools and tcl scripts. Overall, the workflow presented here provides a practical guide for readers to model, simulate, and analyze their own complex biophysical systems involving membranes and nanoplastics.

## Graphical abstract

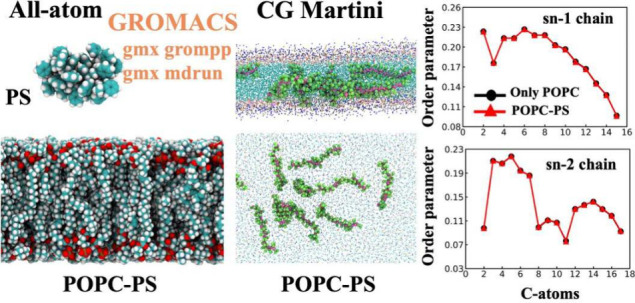

## Introduction

Molecular dynamics (MD) simulations have been used extensively to investigate the biological processes such as protein folding, ligand binding, membrane dynamics, ion transport, and biomolecular interactions.^1–5^ It is a powerful tool that complements experimental observations by offering insights into biomolecular interactions.^6^ These simulations can be conducted at both the atomistic^1,5,7^ and coarse-grained levels.^8,9^ Atomistic simulations provide detailed information about interactions at the atomic scale, whereas coarse-grained simulations simplify molecular representations and reduce the number of degrees of freedom, enabling the study of larger systems over longer time scales. Together, these approaches enable the examination of biochemical processes across a wide range of spatial and temporal regimes.

MD simulations are increasingly used to investigate environmentally relevant molecular systems. One important example is plastic pollution. Over the years, the use of plastics has increased significantly, becoming an essential part of modern life.^10^ However, this extensive use has also raised serious environmental concerns, especially due to the buildup of plastic waste in landfills and oceans.^11–13^ Several studies have shown that plastic nanoparticles can cross the biological barrier, accumulate in aquatic organisms, and induce toxic effects.^14–20^ Understanding these interactions at the molecular level is therefore an important application of MD simulations.

In this tutorial, we present a simple membrane and polystyrene (PS) system to demonstrate the basic workflow for constructing such models using CHARMM-GUI^21^ and performing simulations with GROMACS.^22–24^ We demonstrate both atomistic and coarse-grained MD simulations through six exercises: atomistic MD simulations of (1) PS in water, (2) POPC membrane in water, and (3) POPC–PS complex in water, and coarse-grained MD simulations of (4) PS in water, (5) POPC membrane in water, and (6) POPC–PS complex in water. These exercises use the CHARMM36 force field^25–27^ and the TIP3P^28^ water model for atomistic simulations and the MARTINI2 force field^8^ for coarse-grained simulations. We first describe step-by-step system construction for atomistic simulations using CHARMM-GUI, and for coarse-grained simulations using the polyply^29^ and insane^30^ python scripts. Subsequently, we discuss how to perform the atomistic and coarse-grained MD simulations using GROMACS. In addition, we discuss the use of several built-in GROMACS tools to calculate important biophysical properties of the membrane and nanoplastic. Visualizations of the systems and the trajectories are performed using VMD.^31^

This minimal setup is intended as a pedagogical example and can be extended to study more complex membranes, different types of nanoplastics, and biologically realistic environments. For simulating proteins using GROMACS, the reader can refer to another recently published tutorial.^32^

## Methods

### Prerequisites and Installation

- **CHARMM-GUI**: CHARMM-GUI^21,33–43^ is a web-based platform that provides an intuitive, visual interface for constructing atomistic molecular systems such as nanoplastics, lipid membranes, and protein–ligand complexes. Since it is a web server, you will need to create a free account before using it. No local installation is required.
- **Insane**: A python script for constructing Martini-based membranes and solvent boxes. Note that insane requires setuptools version 81.0.0, whereas installing python via conda may install a newer version (e.g., setuptools 82.0.1), which leads to an error while using insane. A recommended working setup is outlined below:
  (i) (Optional) Create and activate a new conda environment using conda create -n my-env followed by conda activate my-env.
  (ii) Install python in the environment (e.g., conda install python = 3.10).
  (iii) Check the installed version of setuptools using conda list. If it differs from version 81.0.0, downgrade using conda install -c conda-forge setuptools = =81.0.0.
  (iv) Now install insane using pip install insane.
  While we have verified compatibility with setuptools version 81.0.0, other versions may also work, although this has not been systematically tested.
- **Polyply**: A flexible tool for building coarse-grained polymer models and generating topology/coordinate files. Ensure that pip is installed on your system. The dependencies pysmiles and cgsmiles are required to make polyply work, while numba is optional and primarily provides a performance boost. These dependencies can be installed with pip:
pip install pysmiles cgsmiles numba
  Finally, polyply can be installed with pip using
pip install polyply.
  Note that if the environment (my-env) described in the insane section has been created, polyply and its dependencies (pysmiles and cgsmiles) should be installed within the same environment. This ensures that both insane and polyply are accessible from the same environment.
- **GROMACS**: GROMACS is an MD simulation package used for running both atomistic and coarse-grained simulations. It is freely available and can be installed on Linux, macOS, and Windows. Detailed installation instructions are available on the GROMACS manual.^24^ All systems were initially energy-minimized using GROMACS 2021.4 in a local system. Subsequent equilibration and production MD simulations were performed using GROMACS 2022.2 in Syracuse University supercomputing facility. Users running newer versions may encounter warnings arising from updates to the software, particularly related to the thermostat or barostat settings. Readers are advised to review any such warnings carefully before proceeding.
- **VMD**: VMD is used for visualization and basic trajectory analysis. It supports common file formats (e.g., PDB, GRO, XTC) and provides tools for trajectory inspection, structural analysis, and high-quality molecular graphics generation. Installation instructions are available on the VMD Web site.^44^

NOTE: If one does not have access to a supercomputer to run the production steps, they can use a sample output from the Tutorials_files.

### Common File Extensions Used in GROMACS

- .gro or .pdb: Coordinate file that stores atomic coordinates.
- .itp: An “include topology” file containing molecular topology information, including atomic charges, masses, and connectivity of all atoms in a molecule. Typically, each molecule has its own .itp file, and all such files are referenced in the master .top file.
- .top: The master topology file that contains complete system information, including references to all .itp files and the number of each molecule in the system.
- .mdp: MD parameter file. This file specifies how the simulation is carried out, including the length of the run, integration time step, temperature, pressure coupling, and other control parameters. A detailed explanation of the key .mdp parameters is provided in a later section.
- .tpr: A binary run input file that integrates coordinates, topology, force field parameters, and simulation settings specified in the .mdp file.
- .xtc: A trajectory file that stores the coordinates of the system at each time interval specified in the .mdp file.
- .trr: A high-precision trajectory file that stores time, box 150 vectors, coordinates, velocities, and forces. Note that these files 151 are typically very large.
- .edr: An energy file that stores simulation energy data generated during a GROMACS run, such as potential energy, kinetic energy, temperature, pressure, and other thermodynamic properties as a function of simulation time.
- .log: This file contains a detailed record of the simulation. Typically, it includes GROMACS version and compilation information, command-line options used to start the run, input parameters (from the .mdp file), simulation setup details (system size, number of atoms, cutoffs, PME grid, etc.), performance statistics (time step, speed, ns/day, efficiency), and warnings or errors encountered during execution.

### Exercise 1: Perform Atomistic MD Simulations of PS

#### 1.1 Building a PS Chain Using CHARMM-GUI

To investigate the behavior of PS in contact with lipid membranes, it is first necessary to construct an equilibrated PS system in water. As an initial step, we generated a single PS chain comprising 25 styrene monomers and solvated it in water. This model serves as the starting point for subsequent simulations of the polymer–membrane system. The system was built using the CHARMM-GUI Polymer Builder interface,^43^ and the detailed procedure, illustrated with screenshots from the CHARMM-GUI server (Figures S1–S3), is described below.

**(i) Step 1:** From the Input Generator menu, select Polymer Builder

**(ii) Step 2:** In the Polymer Builder interface, set the system type to Solution.

Under the Building Block(s) of Polymer section, select vinyls--polystyrene--atactic as the polymer type in the select unit section. Specify the number of monomer units to be added (in this case, 25). The polymer was capped with −CH_3_ groups at both ends. Click Next Step: Build Polymer Chains in the bottom right corner of the page.

**(iii) Step 3:** Choose the simulation box type (here, a cubic box) and specify the box dimensions. Select water as the solvent. Here, we choose 100 Å in all directions, leading to a 100 × 100 × 100 Å^3^ box. Enter the number of chains, here 1, in the editable box under the #Chain column. Click Next Step: Generate Systems.

**(iv) Step 4:** Use all the default options except in the temperature section, where the temperature is set to 310 K to match the value used in the later simulations. Click Next Step: Generate Equilibrium.

**(v) Step 5:** Click Next Step: Replace into All-atom.

**(vi) Step 6­(a):** In the Input Generation Options section, select the simulation software, here GROMACS. In the Equilibration Input Generation Options section, select the temperature as 310 K.

**(vii) Step 6­(b):** Next, choose *NPT* ensemble in the Dynamics Input Generation Options. Click Next Step: Input Generations. Note that the force field parameter selection is not available for this system in CHARMM-GUI. However, CHARMM-GUI uses the CHARMM36 force field for PS in the water system by default.

During this step, the system is equilibrated in the background using the CHARMM package. After the process is completed, input files for GROMACS are generated, including the structure files, force-field parameter files, topology files, and .mdp files required for GROMACS simulations.

**(viii) Step 7:** Click Download.tgz in the top right corner of the page to download the generated files. These include the PDB, PSF, topology and parameter files, and simulation input configuration files.

A schematic diagram of system construction using CHARMM-GUI is shown in Figure 1(a), while the molecular representation of the PS in water built from styrene is shown in Figure 1(b)–(d).

**Figure 1:**
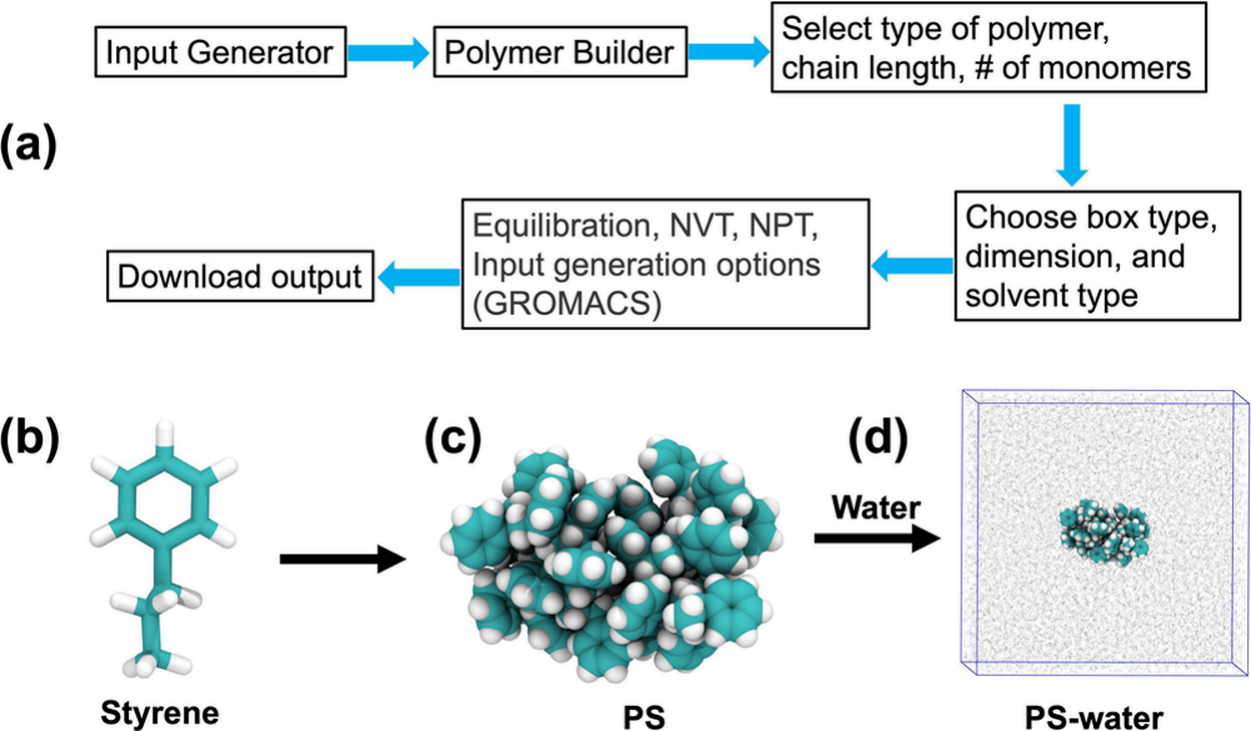
(a) Schematic diagram of building PS in water using CHARMM-GUI. Atomistic model of (b) a single styrene monomer, (c) PS made of 25 monomers of styrene, and (d) PS in a simulation box filled with water.

Within the downloaded directory, navigate to the gromacs subfolder. This directory contains the essential system input files, including step3_input.gro and topol.top, and the corresponding .mdp parameter files. The force field parameter files forcefield.itp, S1P1.itp, and TIP3.itp are located in the gromacs/toppar subdirectory. For consistency across this tutorial and to avoid naming conflicts commonly encountered with files generated by CHARMM-GUI, step3_input.gro was renamed to PS-water.gro, and forcefield.itp was renamed to forcefield_PS.itp. It is important to note that all the .mdp input files used throughout the exercises were not directly adopted from CHARMM-GUI. Rather, the .mdp files were directly downloaded from the GROMACS tutorial (for example, Lysozyme in Water),^45^ with modifications applied for specific requirements of our simulation systems. The contents of these files are discussed below.

- PS-water.gro contains coordinates of the solvated system (PS and water).
- S1P1.itp contains information such as charges, masses, and connectivity between the atoms of the PS molecule.
- TIP3.itp includes information such as charges, masses, and connectivity between the atoms of the TIP3P water molecules.
- forcefield_PS.itp is a topology file containing charges and masses of each atom type and the parameters for all bonded and nonbonded interactions of the system.
- topol.top is the master topology file that specifies the paths to all the above .itp files and defines the number of each molecule in the system.

The structure of the topol.top file is given below.
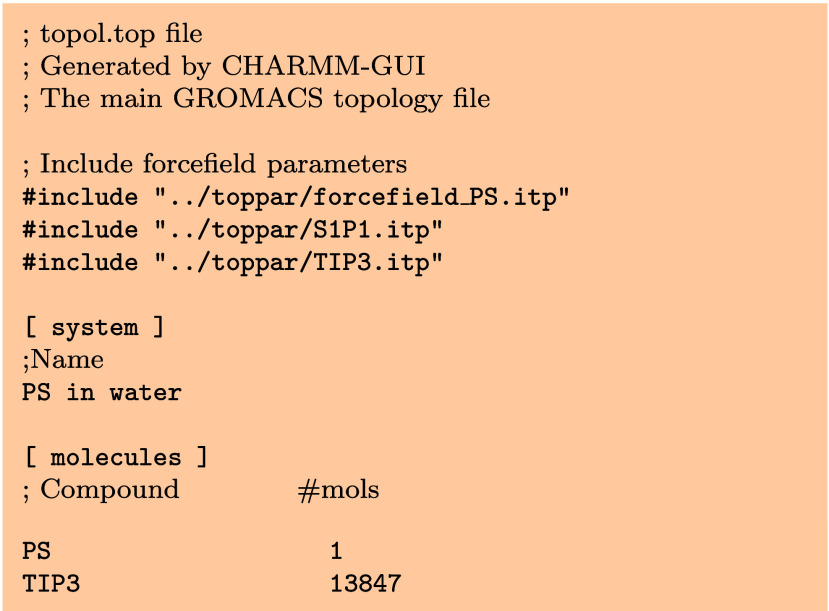


The topol.top file is provided in the directory Tutorials_files/1.Atomistic_simulations/1. PS_in_water, while the topology and force field files are located in the toppar directory under Tutorials_files/1. Atomistic_simulations. All steps described in the following sections of this exercise were executed from the directory Tutorials_files/1.Atomistic_simulations/1.PS_in_water. Ensure that all subsequent commands of this exercise are run from the same directory. Accordingly, the paths to the .itp files in the “Include force field parameters” section were adjusted to reflect this directory structure. The text following a semicolon (;) serves as a comment. The [system ] section defines the system name, which can be chosen arbitrarily. In this exercise, we define it as PS in water. The final section, [molecules], lists all molecular species in the simulation along with their respective counts. In our case, PS and TIP3 represent polystyrene and water, respectively. Having prepared all the necessary files, we are now ready to proceed with energy minimization and MD simulations using GROMACS.

Note that the MD parameter files used in energy minimization, *NVT* equilibration, *NPT* equilibration, and the production MD simulation were denoted as minim.mdp, nvt.mdp, npt.mdp, and md.mdp, respectively, following the naming convention used in the tutorial and these designations are maintained consistently throughout all subsequent exercises.

While CHARMM-GUI is a widely used tool for constructing a wide range of biomolecular systems, it is important to note that some polymeric systems, such as cross-linked polymers or unique functionalized polymers,^46,47^ cannot be directly generated within this framework. Modeling such systems would require specialized iterative approaches.^48–51^ In this introductory tutorial, we only focus on standardized systems that can be readily built using CHARMM-GUI.

#### 1.2 Energy Minimization

CHARMM-GUI generated the required structure and topology files for MD simulations. Consequently, we did not perform the manual topology generation and solvation steps using GROMACS. We proceed directly to the energy-minimization stage. During system setup, molecules are initially placed at random within the simulation box, which can lead to unfavorable interatomic contacts. To resolve these steric clashes and obtain a stable starting configuration, we perform energy minimization. Note that energy minimization does not guarantee convergence to the global energy minimum of the system. However, it ensures a reasonable and physically acceptable structure suitable for MD simulations. For this step, we use the files PS-water.gro, topol.top, and minim.mdp. An example of the minim.mdp file is shown below. This file specifies the minimization method (here, steepest descent), the maximum number of minimization steps, the force convergence criterion, and other related parameters. A brief explanation of each keyword in minim.mdp file is provided alongside the file.
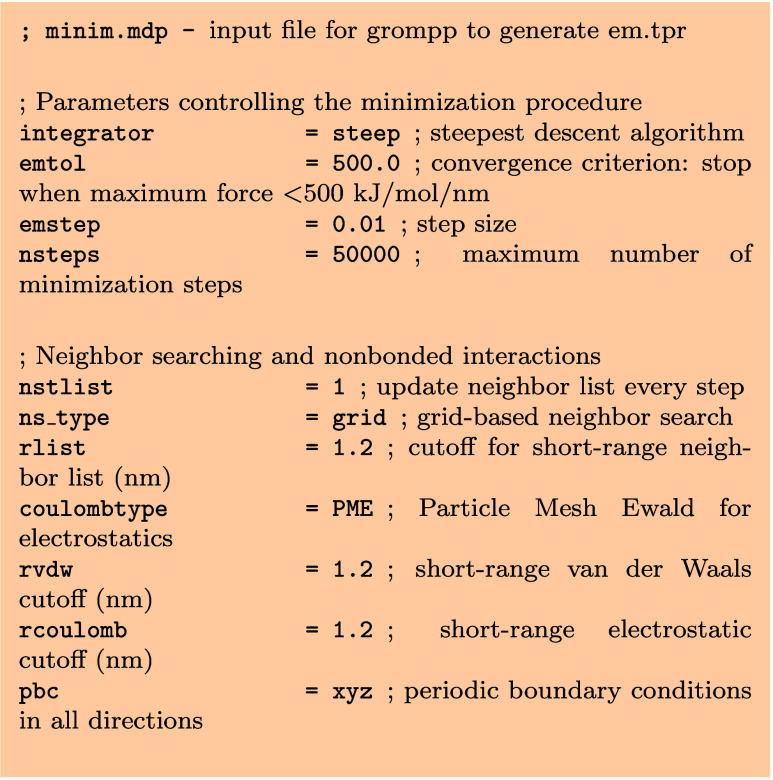


Energy minimization in GROMACS is performed using the following commands:
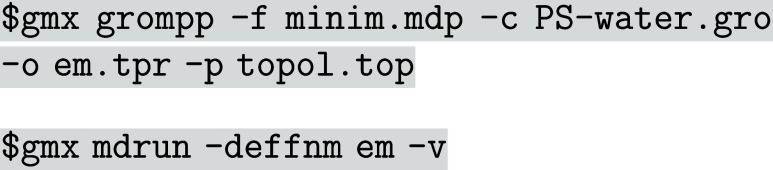


The GROMACS preprocessor (gmx grompp) reads the topology (topol.top), structure (PS-water.gro), and MD parameter (minim.mdp) files, and combines them into a portable run input file (em.tpr). This em.tpr file serves as the sole input for gmx mdrun. The mdrun program can perform a variety of tasks, including energy minimization, MD simulations, normal-mode analysis, and stochastic dynamics. The -deffnm option specifies a common prefix for all input and output files associated with the run. The optional -v flag prints detailed information, including the step number, potential energy, temperature, and other quantities on the terminal.

After executing the above commands, the progress of the minimization can be followed in the terminal output, and a detailed log is stored in em.log. The potential energy as a function of minimization steps is recorded in the binary energy file (em.edr). To extract the potential energy, the following command is used:
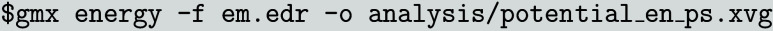
Running the above command displays a list of properties, including energy components, pressure, temperature, and others. To extract the potential energy, select the corresponding option from the list (option 9 in our case). After making the selection, press 0 to exit. The potential_en_ps.xvg is saved inside the Tutorials_files/1.Atomistic_ simulations/1.PS_in_water/analysis directory along with the jupyter notebook file (Analysis.ipynb) for the plot of potential energy is provided in the directory Tutorials_files.

The potential energy profile as a function of simulation steps is shown in Figure 2(a). The curve converges smoothly to a stable negative value, confirming that the system has reached an energy minimum and is ready for equilibration.

**Figure 2:**
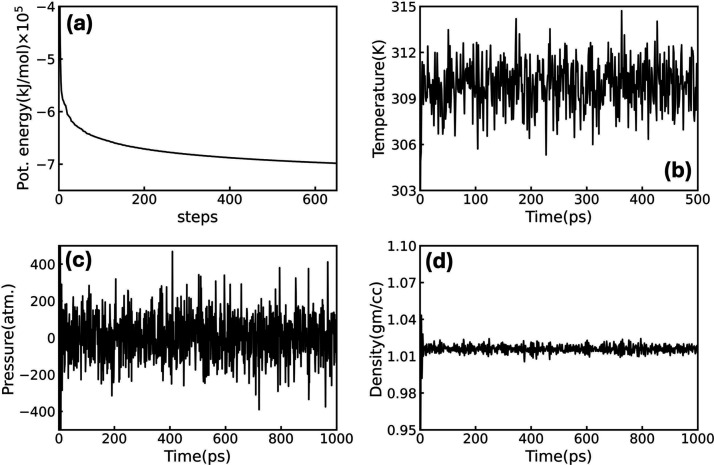
(a) Potential energy of the system as a function of simulation steps. (b) Temperature, (c) pressure, and (d) density of the system as a function of simulation time.

#### 1.3 MD Simulations

##### 1.3.1 *NVT* Equilibration

Energy minimization provides a reasonable starting structure for MD simulations. However, prior to data collection, thermodynamic variables such as temperature, pressure, and system density must be equilibrated to the target ensemble. We perform equilibration in two sequential steps: *NVT* (constant number of atoms, volume, and temperature) and *NPT* (constant number of atoms, pressure, and temperature) equilibration.

We begin with *NVT* equilibration using the minimized structure (em.gro) obtained from the previous step. The system contains three primary groups based on residue names: STYRR and STYRS for polystyrene (PS) and TIP3 for water. Since STYRR and STYRS belong to the same molecule (PS), they are combined into a single group by creating an index.ndx file. This grouping is achieved using the gmx make_ndx command:
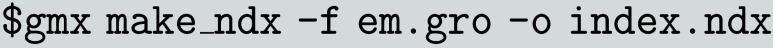
After executing this command, the output appears as follows:
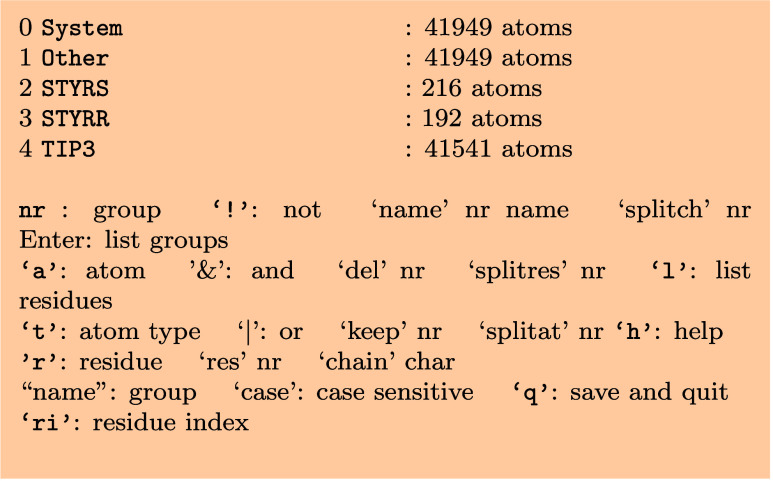


In our case, groups 2 and 3 (PS residues) are combined. Pressing q saves and exits the program (see below).
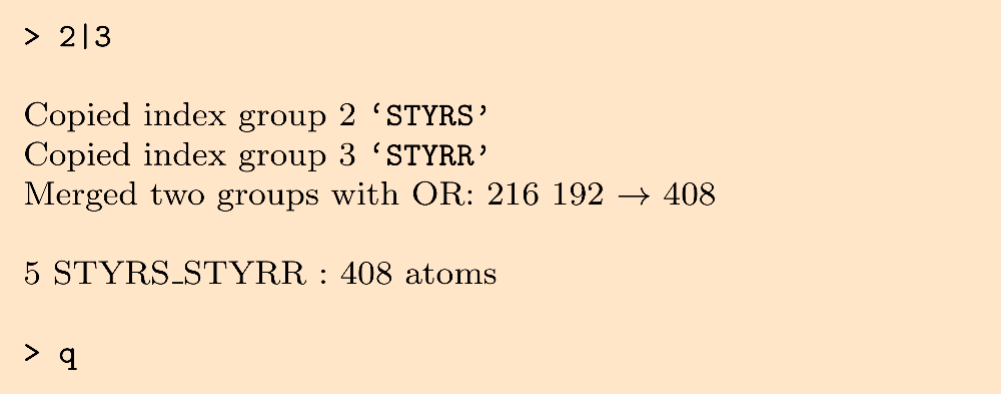


The newly generated index.ndx file can be visualized using the following command:
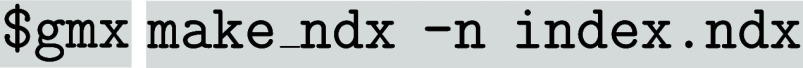
The output appears as follows:
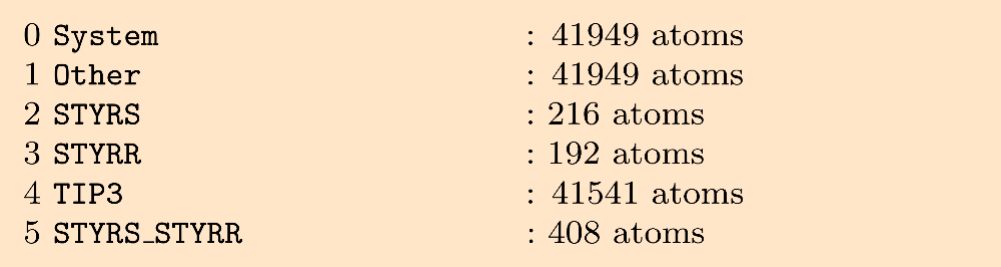


We will use the STYRS_STYRR group in the nvt.mdp file, which is the input parameter file for *NVT* equilibration. Note that the index.ndx file generated in this step is only required when groups are combined, and the resulting group is used in the coupling section of the .mdp file, provided that this group is not available by default. The resulting index.ndx file is used as input for subsequent gmx grompp commands. The nvt.mdp file (shown below) specifies simulation parameters, including position restraints applied to the heavy (carbon) atoms of PS using the -DPOSRES option.
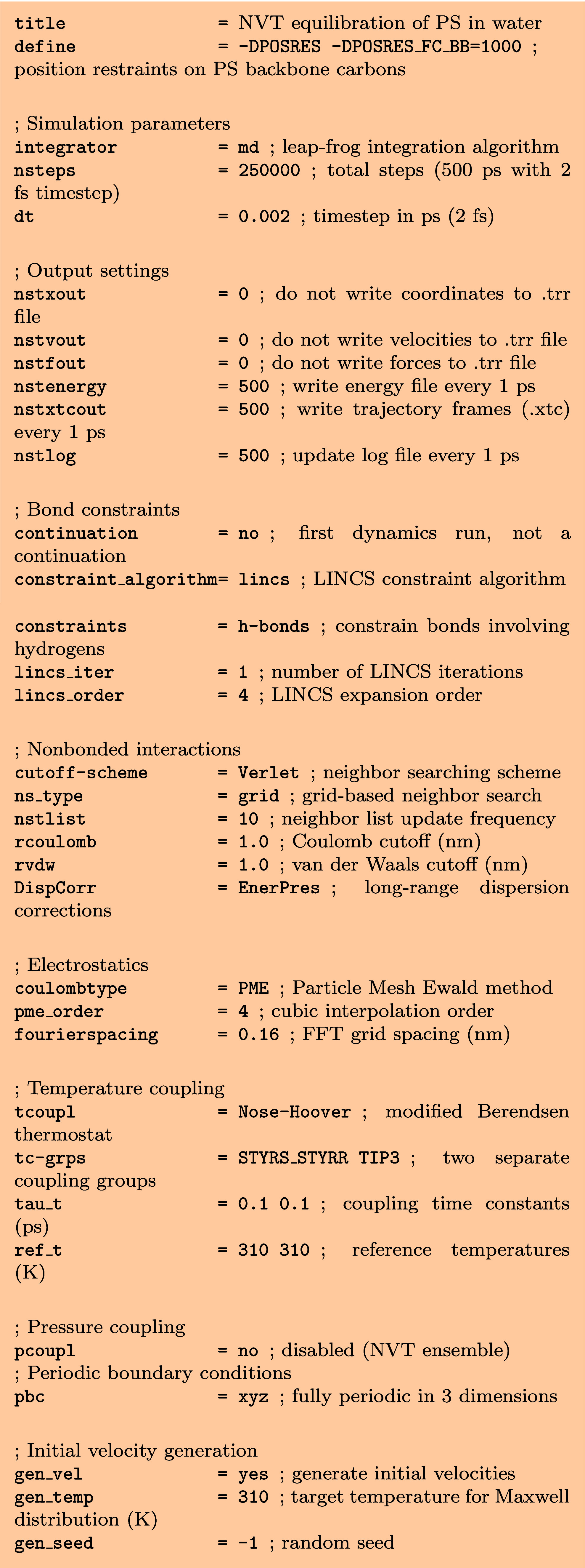
A brief description of each keyword in the nvt.mdp file is provided alongside the input. For instance, the number of simulation steps is set to 250000, with a time step of 2 fs, corresponding to a total simulation time of

nsteps × dt = 250000 × 0.002 = 500 ps

The POSRES for PS is defined at the end of the S1P1.itp file in the excerpt given below.
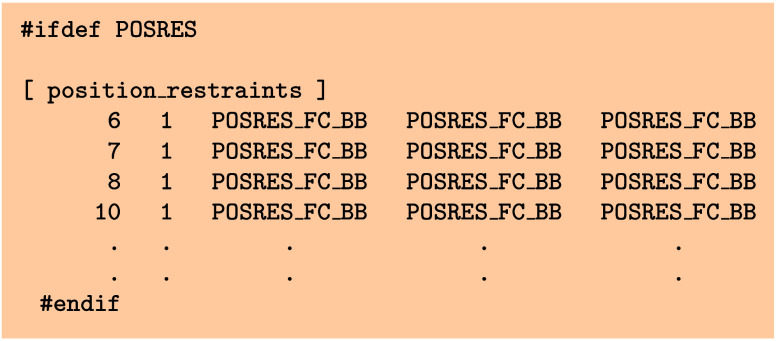
The numbers 6, 7, 8, 10, and so on represent the atom numbers of C atoms since the C atoms are restrained here. The value of POSRES_FC_BB (=1000 kJ/mol) is defined in the nvt.mdp file. LINCS^52^ constraint algorithm is used to constrain the bonds involving H atoms. The electrostatic interactions are calculated using the particle mesh Ewald (PME)^53,54^ method. The temperature coupling is performed using the Nose–Hoover thermostat.^55,56^ The *NVT* equilibration is performed using the following commands:
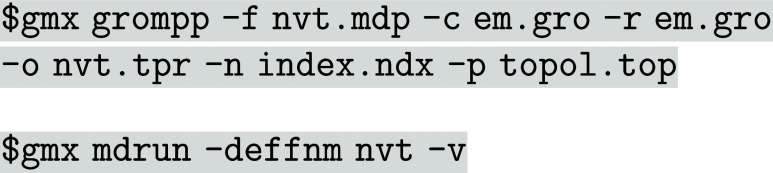
We use em.gro as the input coordinate file for *NVT* equilibration, and the -r option specifies the restraint file. In this case, the same em.gro file is provided for -r, since GROMACS2018 and later require this option. Note that the -r option is required when restraints are enabled in the .mdp file. Adding the optional -v flag to the gmx mdrun command displays the estimated completion time of the job. However, when running jobs on a supercomputing cluster, it is generally not recommended to include the -v option.

The system temperature during equilibration can be monitored by extracting it from the *NVT* energy (nvt.edr) file:
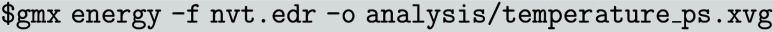
Choose the appropriate option for temperature (15 here) and hit 0 to exit. As shown in Figure 2(b), the temperature quickly stabilizes at the target value from the beginning of the simulation, with only minor fluctuations, indicating thermal equilibrium. The jupyter notebook for the plot is also provided in the Tutorials_files directory.

##### 1.3.2 *NPT* equilibration

Now we perform an *NPT* equilibration from the last step of the *NVT* equilibration. The *NPT* parameter file (npt.mdp) is very similar to the *NVT* parameter file, except for the pressure coupling settings, which are enabled to allow the system volume to adjust. The pressure-coupling section below highlights this difference.
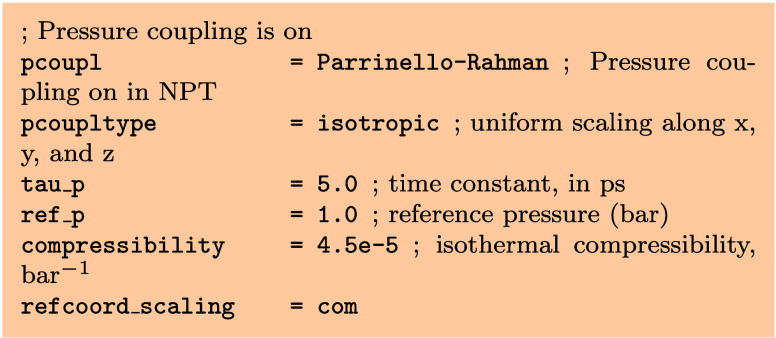
Here, the Parrinello–Rahman^57^ pressure coupling method was used, although the Berendsen^58^ method can also be applied. The *NPT* equilibration is performed using the following commands:
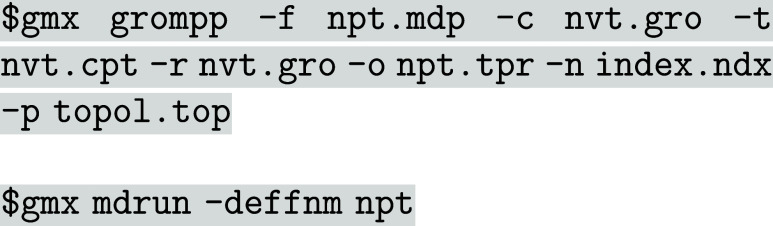
Here, gmx grompp generates the run input file (npt.tpr) using the equilibrated coordinates from the *NVT* step (nvt.gro) and the corresponding checkpoint file (nvt.cpt) to continue the simulation. The mdrun command executes the *NPT* simulation, with -deffnm defining the common prefix for input and output files. After *NPT* equilibration, the system pressure and density were monitored throughout the simulation. These properties can be extracted from the energy file (npt.edr) using the gmx energy tool:
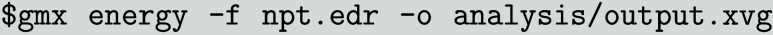
Upon executing this command, an interactive window appears asking for the parameter to calculate. We first selected the group corresponding to pressure (by selecting 16 and entering 0 to exit) and saved the output as pressure_ps.xvg. The command was then run again, this time selecting the group corresponding to density. In this case, select 22, followed by entering 0 to exit, and the output was saved as density_ps.xvg. The files pressure_ps.xvg and density_ps.xvg are provided in the following directory: Tutorials_files/1.Atomistic_ simulations/1.PS_in_water/analysis. The jupyter notebook file (Analysis.ipynb) for these plots is provided in the directory Tutorials_files. As shown in Figure 2(c),(d), the system pressure and density fluctuate around the target value throughout the simulation, with only minor deviations.

##### 1.3.3 Production MD Simulations

After equilibration, the system has reached the target temperature and pressure as specified in the parameter files. At this stage, the position restraints on the PS carbon atoms are removed, and the *NPT* simulation is continued. This step is used to collect data during the production phase of the simulation. The simulation parameters for this stage are defined in the md.mdp file, available in the Tutorials_files directory. This file is similar to npt.mdp, except that position restraints are removed. Additionally, the production run is set for 400 ns with a time step of 2 fs. The production steps in all exercises may require the use of a supercomputing cluster. The simulation is launched using the following commands:
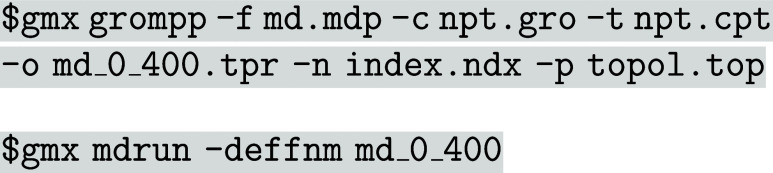
Here, gmx grompp prepares the input md_0_400.tpr file using the equilibrated coordinates (npt.gro and npt.cpt), and the system topology (topol.top) from the *NPT* equilibration. The gmx mdrun command executes the production simulation, with -deffnm defining the common prefix for all input and output files. At the end of each simulation, GROMACS writes the final .gro (md_0_400.gro) file of the system along with the trajectory (md_0_400.xtc), log (md_0_400.log), and related output files. Note that a short trajectory file (md_0_400_noPBC_short_trj.xtc) containing the entire system is provided in the directory: Tutorials_files/1.Atomistic_simulations/1.PS_in_water. One can also extract the final structure, or any structure at a chosen time point, from the trajectory using
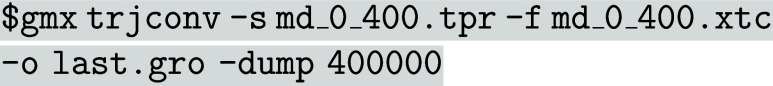
Here, the value provided after -dump is the time (in ps) corresponding to the frame to be written. For example, -dump 400000 extracts the structure at 400 ns.

##### Restart a Simulation

After the initial 400 ns simulation, the trajectory can be extended for an additional simulation (e.g., 400 ns) using the gmx convert-tpr command:
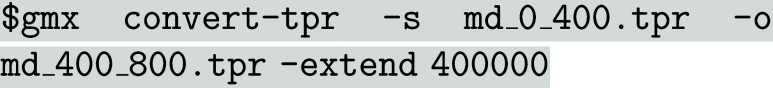
The -extend option specifies the additional simulation time in picoseconds. To continue the simulation from the previous run, the corresponding checkpoint file (md_0_400.cpt) must be provided to mdrun, since it contains the details of the last simulation step. The extended run is then executed as
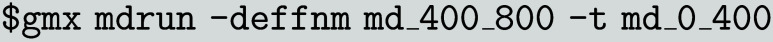
Again, the filename (md_400_800) specified after the -deffnm option defines the common prefix for all input and output files, while the filename (md_0_400) specified after the -t option refers to the checkpoint file (md_0_400.cpt), containing the latest coordinates and velocities of the system. Note that GROMACS does not need the file extensions. This procedure allows the production simulation to continue while preserving velocities, positions, and other simulation variables. After completion of the simulations, corresponding output files are generated with the base name md_400_800.

At this stage, we have two trajectories (md_0_400.xtc and md_400_800.xtc), each corresponding to 400 ns of simulation, totaling 800 ns. These two trajectory files can be concatenated into a single trajectory file, enabling analyses to be carried out on a single continuous trajectory. The concatenation was performed using the gmx trjcat tool:
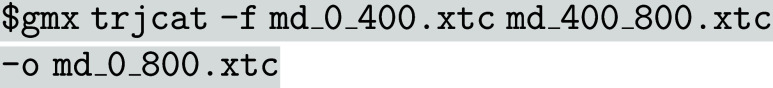
This produces a single, full trajectory file, md_0_800.xtc. To visualize the trajectory in VMD, first load any .gro (e.g., npt.gro) file from the system and then load the md_0_800.xtc file on top of the loaded structure. Note that in this exercise, the trajectory file md_0_400.xtc was used for the analysis of system properties in the following section. Therefore, output files corresponding to the restart simulation (400–800 ns) are not provided. This step is included solely to illustrate the procedure for restarting a simulation.

#### 1.4 Analysis

It is necessary to correct for periodic boundary conditions (PBC) because during the simulations, parts of a molecule can move outside the simulation box and re-enter from the opposite side. To account for this, we apply PBC corrections using the gmx trjconv command:
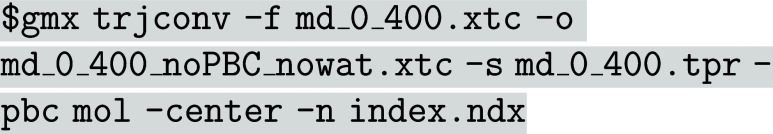
Executing this command opens an interactive window displaying all groups in the system. We selected the PS molecule (resname STYRS_STYRR) (group 5, see the index.ndx file) as the group to center the box, and chose the same group (STYRS_STYRR) as the output group. The options -pbc mol and -center ensure that each molecule is made whole and centered in the simulation box. A detailed description of these options can be found in the GROMACS documentation for gmx trjconv. Note that the trajectory file (md_0_400_noPBC_nowat.xtc) contains only PS. Water molecules were removed because the analysis focuses on the properties of PS. To load this trajectory file in VMD, a .gro file containing only PS (STYRS_STYRR) must first be generated so that it matches the contents of the water-free trajectory. The water-free .gro file can be generated using VMD or using gmx trjconv command. The trajectory file can then be loaded on top of this newly created .gro file (npt_nowat.gro). In addition, we generated a water-free .tpr (md_0_400_nowat.tpr) file, which is used together with the corresponding water-free trajectory (md_0_400_noPBC_nowat.xtc) for analyzing system properties. This file is created using the following command:
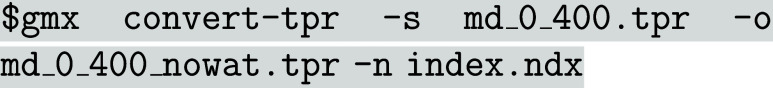
Executing this command shows the output appears below:
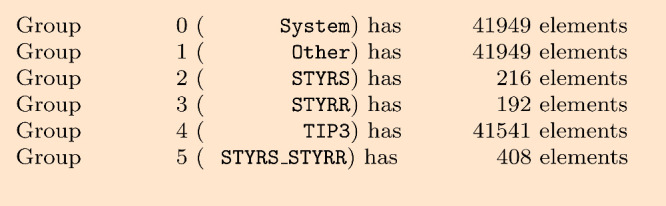


Group 5 was selected to generate the water-free .tpr file. A corresponding water-free index file was then created by removing all groups containing water using the command
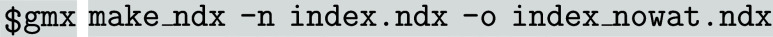
Executing this command produces the following output:
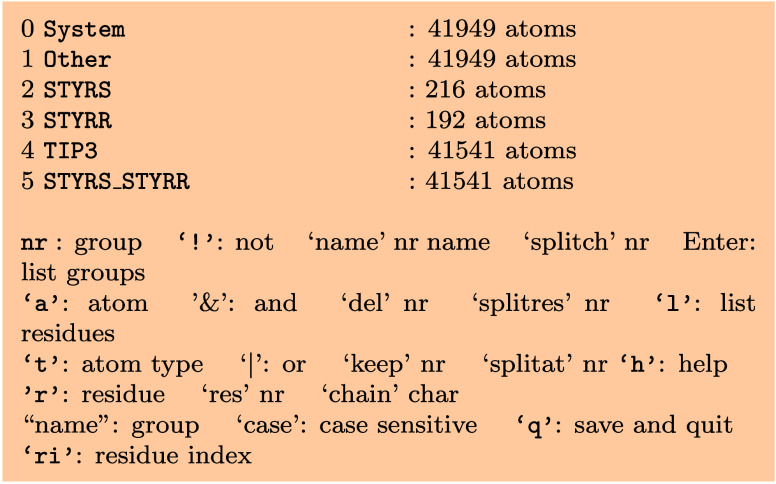


The groups 0, 1, and 4 containing water were removed using the del command, and the updated index file (index_nowat.ndx) was saved using the q command in the interactive window, as shown below:
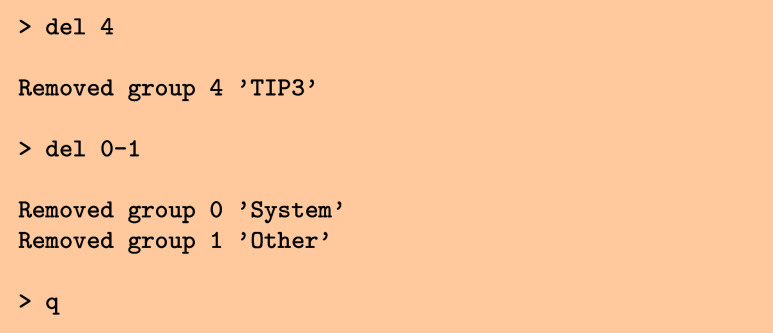


Note that in the gmx make_ndx interactive session, groups were deleted sequentially from the end to avoid index shifting issues. Since group indices are automatically renumbered after each deletion, removing groups in reverse order ensures that the intended group numbers remain valid throughout the process.

At this stage, we have the water-free trajectory file (md_0_400_noPBC_nowat.xtc), the water-free .tpr file (md_0_400_nowat.tpr), and the water-free index file (index_nowat.ndx). These three files are then used as input for subsequent analysis of system properties using GROMACS tools, as described in the following section.

##### 1.4.1 Radius of Gyration of PS

The radius of gyration (*R*_*g*_) is a structural property that provides insight into the overall compactness of a molecule. A lower *R*_*g*_ value indicates a more compact or folded structure, whereas a higher *R*_*g*_ suggests that the molecule is more extended or unfolded. *R*_*g*_ was calculated using the following equation:

1 $$R_{g}=\sqrt{\frac{1}{N}\overset{N}{\underset{i=1}{\sum }}{\left|r_{i}-r_{\mathrm{COM}}\right|}^{2}}$$

where *N* is the number of atoms (or coarse-grained beads), **r**_*i*_ is the position vector of the *i*-th atom, and **r**_COM_ is the position vector of the center of mass of PS.

The *R*_*g*_ of the PS nanoparticle was computed using the gmx gyrate module available in the GROMACS analysis suite. The command to calculate *R*_*g*_ of PS using gmx:
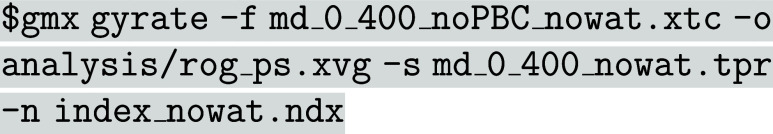


In the interactive window, the group corresponding to STYRS_ STYRR was selected to calculate the *R*_*g*_ of PS. The final PS structure from a 400 ns PS–water simulation is displayed in Figure 3(a), where PS is enclosed by a circle, and the *R*_*g*_ is indicated by an arrow representing the radius of the circle. The *R*_*g*_ converges after 200 ns of simulations (Figure 3(b)). The average *R*_*g*_ over the converged time interval is 7.8 Å.

**Figure 3:**
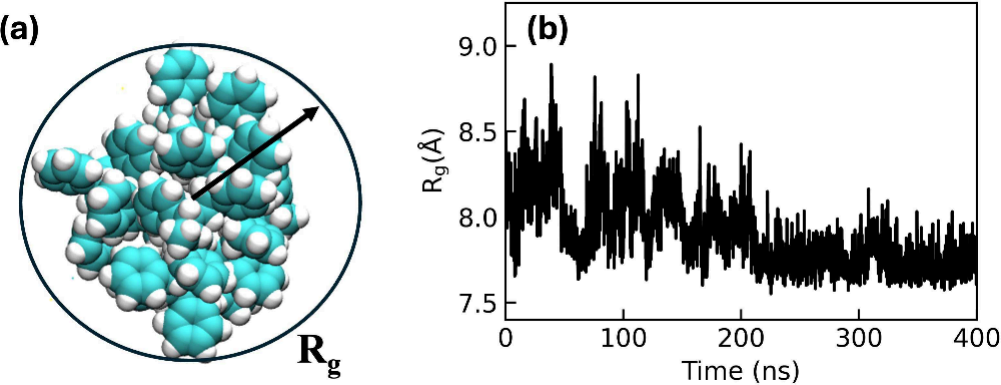
(a) Molecular representation of PS illustrating the radius of gyration (*R*_*g*_) and (b) *R*_*g*_ value during the course of the simulation.

### Exercise 2: Perform Atomistic MD Simulations of a Model Membrane

#### 2.1 Building POPC Membrane Using CHARMM-GUI

To explore the structural and dynamical properties of membranes, it is essential to begin with a well-equilibrated membrane model. In this section, we describe the construction of a POPC membrane system, which serves as the reference membrane for subsequent simulations of PS–membrane system. We built a membrane composed of 200 POPC molecules per leaflet, solvated with water, and added 0.15 M NaCl. The system was constructed using the CHARMM-GUI Membrane Builder.^35–42^ The step-by-step procedure is outlined below, while representative screenshots from the server are provided in Figures S4–S6.

**(i) Step 1:** From the Input Generator menu, select Membrane Builder.^35–42^

**(ii) Step 2:** Choose the option Membrane Only System to build a bilayer without any embedded proteins. Click Next Step: Select Lipids in the bottom right of the page.

**(iii) Step 3:** Choose Heterogeneous Lipid and Rectangular as the box type, and set the water thickness to 50 Å. Click on the Numbers of lipid components option. Specify the lipid type and number (here, 200 POPC lipids in each leaflet). Clicking Show the system info displays the estimated areas and the number of lipids in the upper and lower leaflets, including the box lengths along the *x* and *y* directions. Next, click Next Step: Determine the System Size.

**(iv) Step 4:** In the System Building Options section, choose the Replacement method. Next, set Distance as the Ion Placing Method. Select NaCl as the Basic Ion Types with a concentration of 0.15 M. Click Calculate Solvent Composition. This displays the number of Na^+^ and Cl^–^ ions that will be added to the system. Finally, click Next Step: Build Components.

**(v) Step 5:** In this step, the bilayer is generated. Click Next Step: Assemble Components to generate ions and the water box.

**(vi) Step 6:** Again click Next Step: Assemble Components. This step assembles all components (lipids, water, and ions).

**(vii) Step 7:** In the Force Field Options section, select the force field, here CHARMM36m. In the Input Generation Options section, select the simulation software, here GROMACS. In the Equilibration Options section, select Generate grid information for PME FFT automatically, choose the ensemble type, for example, *NPT* or *NVT*, here *NPT*, and set the temperature to 310 K. Click Next Step: Generate Equilibration and Dynamics Inputs.

**(viii) Step 8:** Click Download.tgz in the top right corner of the page to download the generated files. These include the PDB, PSF, topology, and parameter files, as well as simulation input configuration files.

This is illustrated in Figures S4–S6 with corresponding snapshots, while the molecular representation of the POPC–water built from a single POPC is shown in Figure 4.

**Figure 4:**
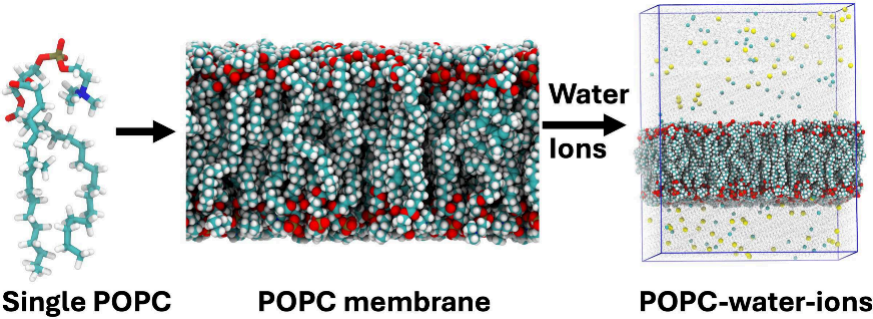
(Left) Single POPC molecule, (middle) POPC membrane consists of 200 POPC molecules in each leaflet, and (right) POPC membrane in water and ions. Sodium and chloride ions are illustrated in yellow and cyan spheres, respectively.

To perform the simulations, several files were obtained from CHARMM-GUI in the gromacs folder; among these, the essential files required for running the simulations include step5_input.gro, topol.top, toppar/forcefield.itp, toppar/POPC.itp, toppar/SOD.itp, toppar/CLA.itp, and toppar/TIP3.itp. The files step5_input.gro and toppar/forcefield.itp were renamed as POPC-water.gro and toppar/forcefield_POPC.itp, respectively. The files POPC-water.gro and topol.top were copied to the directory Tutorials_files/1.Atomistic_ simulations/2.POPC_in_water, while all .itp files were copied to Tutorials_files/1.Atomistic_ simulations/2.POPC_in_water/toppar directory. The .mdp files used in this exercise were obtained from the GROMACS tutorial on Membrane Protein^59^ and modified as described in the following sections. The structure file POPC-water.gro defines the full system coordinates. The force field files include POPC.itp, which contains information such as atomic charges, masses, and connectivity for a single POPC molecule, and forcefield_POPC.itp, which contains information including the bonded and nonbonded parameters for POPC. The topol.top file links all the required .itp files and defines the number of each molecular species present in the system. The topol.top file appears as below:
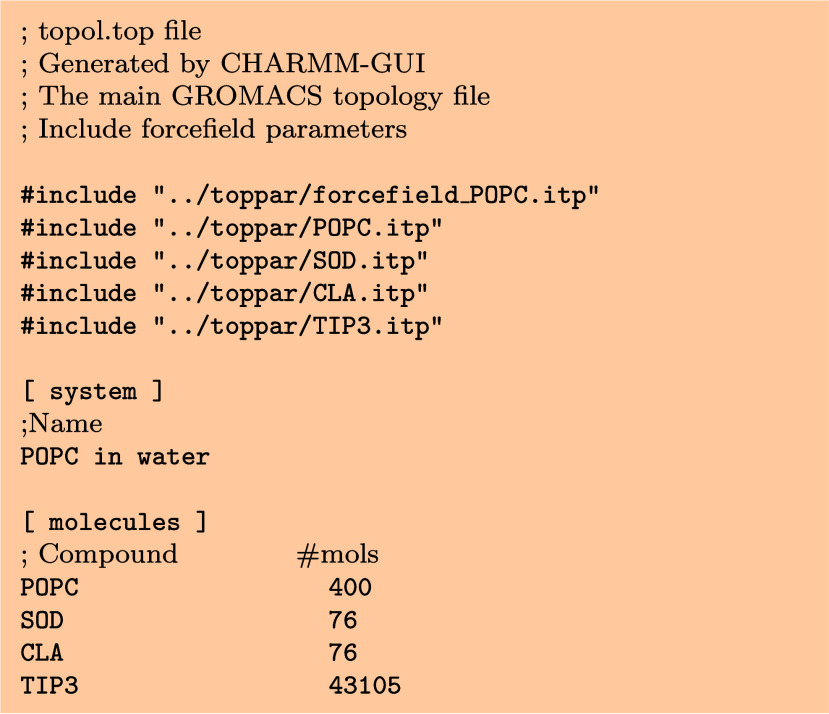


All steps described in this exercise should be carried out from the directory Tutorials_files/1.Atomistic_ simulations/2.POPC_in_water; ensure that you are working within this directory throughout this exercise. The overall procedure, including energy minimization, equilibration (*NVT* and *NPT*), and production MD simulations, follows the same steps as described previously. The main difference arises from the membrane anisotropy, which requires modification of the pressure-coupling scheme. Note that all the gmx grompp and gmx mdrun commands are identical to those in the previous exercise.

#### 2.2 Energy Minimization

The em.mdp file to perform energy minimization is identical to that in Exercise 1. Energy minimization was carried out using the following GROMACS commands:
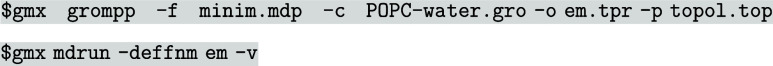
This run generates an energy file (em.edr), from which the potential energy of the system was extracted. As shown in Figure 5(a), the potential energy converges, indicating successful energy minimization.

**Figure 5:**
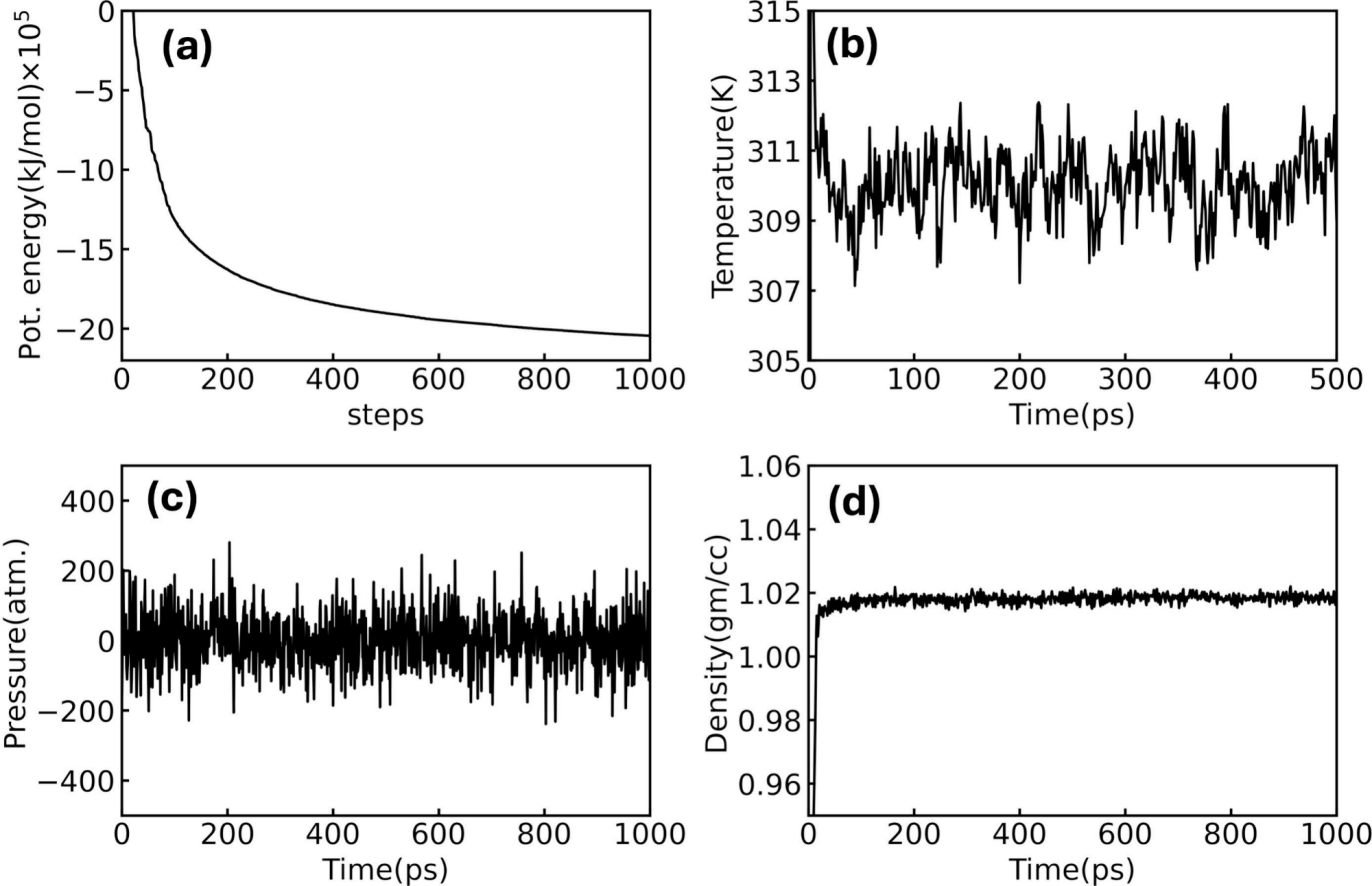
(a) Potential energy of the system as a function of simulation steps, (b) temperature, (c) pressure, and (d) density of the system over the course of the simulation.

#### 2.3 Creating an Index File

An index.ndx file was created using the structure file (em.gro) obtained from the energy minimization step. Alternatively, the initial structure file (POPC-water.gro) can also be used at this stage. The same procedure as in Exercise 1 was followed to generate the index.ndx file using the command:
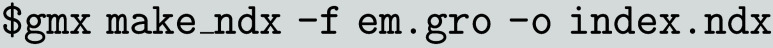
After executing this command, the output appears as follows:
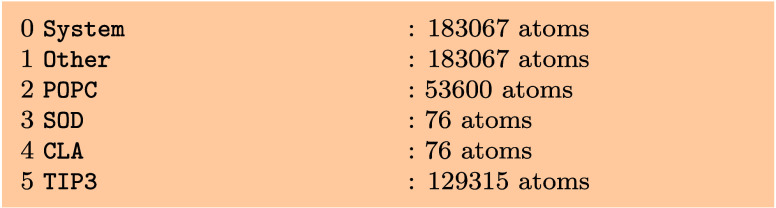


Groups 3, 4, and 5 were combined, as well as groups 2, 3, and 4, using the following procedure.
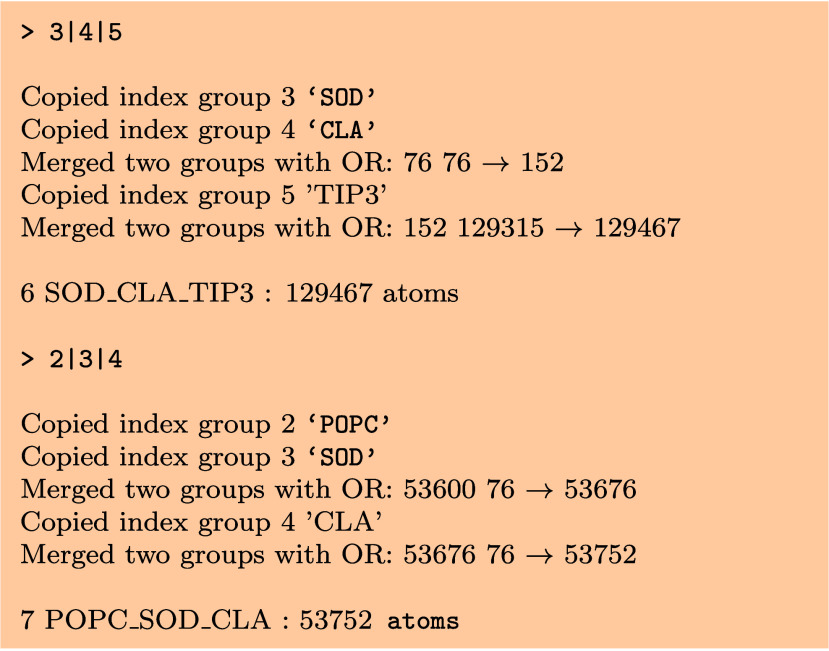


To confirm that the above groups are created in the index.ndx file, execute the command $gmx make_ndx -n index.ndx. The output appears as follows:
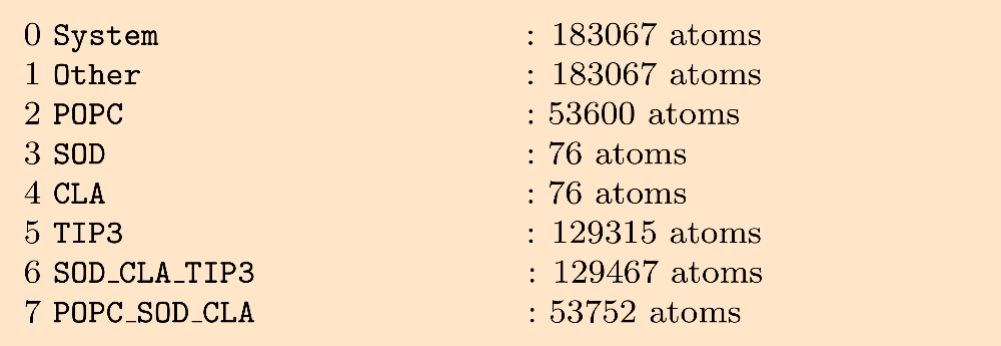


The group SOD_CLA_TIP3 created above is used during the equilibration (*NVT* and *NPT*) and production run stages, while the group POPC_SOD_CLA is used in the analysis section, discussed later.

#### 2.4 *NVT* Equilibration

The nvt.mdp file is similar to that in the previous exercise except that the membrane simulation needs center of mass (COM) motion removal. So, a new section is added in the nvt.mdp file that treats COM motion removal. In interfacial systems, such as membrane–water, the membrane and solvent often exhibit lateral drift. To account for this, the COM of the membrane and that of the solvent must be corrected independently. Without separate treatment, the two phases may drift in opposite directions, leaving the total system COM unchanged while introducing artificial relative motion. The COM motion removal block is shown below:
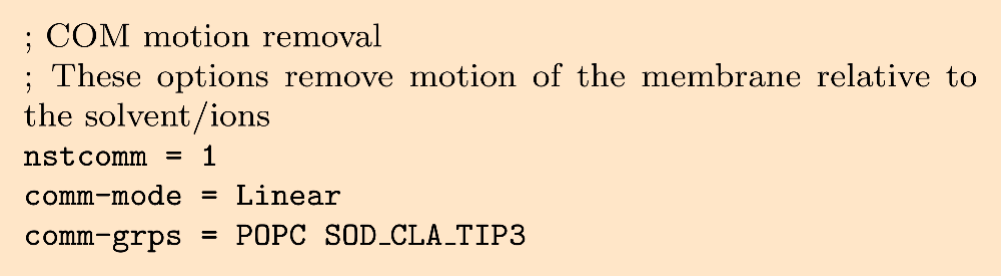


Note that the comm-grps here are set to POPC and SOD_CLA_TIP3, and the tc-grps defined in the temperature coupling section of the nvt.mdp file in Exercise 1 have also been modified to POPC and SOD_CLA_TIP3.

During the *NVT* equilibration, positional restraints were applied to the phosphorus atoms of the POPC lipids by including define = -DPOSRES -DPOSRES_FC_LIPID = 1000 in the nvt.mdp file. The POSRES and POSRES_FC_LIPID keywords are defined at the end of the POPC.itp file. This file is provided in the directory Tutorials_files/1.Atomistic_simulations/ toppar. The *NVT* equilibration was performed using the same commands as those used in Exercise 1.
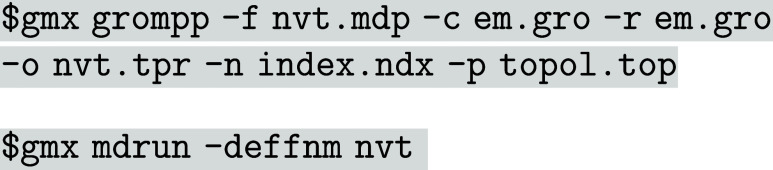


To assess the equilibration of the system, we calculated the temperature from the nvt.edr file, following the same procedure as in Exercise 1. The file is provided in the following directory: Tutorials_files/1.Atomistic_simulations/2. POPC_in_water/analysis/equilibration_ parameters. Figure 5(b) shows the temperature of the system over time. The temperature has converged, indicating that the system reached equilibrium.

#### 2.5 *NPT* Equilibration and Production Run

The npt.mdp file is similar to the nvt.mdp file described above, including the section for COM motion removal. A key difference from the npt.mdp used for the PS–water system is that this file employs semi-isotropic pressure coupling rather than isotropic coupling. This is particularly important for membrane systems because semi-isotropic pressure coupling allows the box vectors in the *xy* plane to fluctuate together while treating the *z* dimension independently, thus preserving realistic membrane mechanics. Otherwise, lipid bilayers can compress or stretch easily in the lateral (*xy*) plane but exhibit limited flexibility in the bilayer normal (*z*) direction. An isotropic pressure coupling would be inappropriate because it would artificially distort the bilayer thickness, yielding unphysical results.

The pressure coupling block defined in npt.mdp file is shown below:
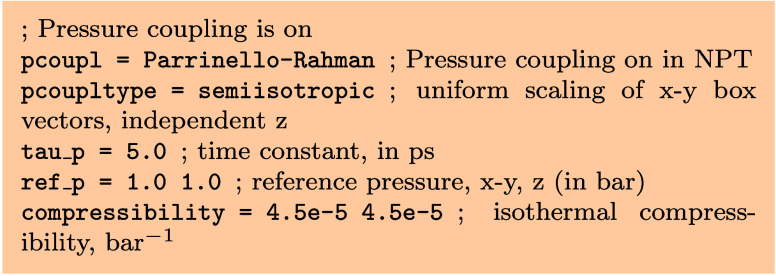


The *NPT* equilibration is performed using the following commands:
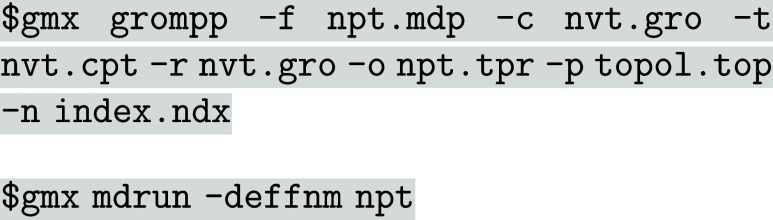


Pressure and density are calculated as before, using the energy file npt.edr. These files are provided in the following directory: Tutorials_files/1.Atomistic_simulations/ 2.POPC_in_water/analysis/equilibration_ parameters. As shown in Figure 5(c),(d), the system reaches the desired pressure and density.

Once equilibrated, the simulation proceeds to the production stage, where membrane-specific observables such as membrane thickness, area per lipid, and lipid order parameter can be extracted. The production run simulation is performed with the commands shown below:
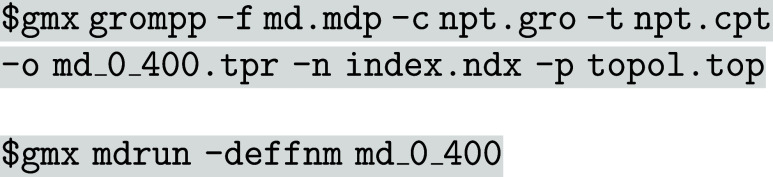
Here, a 400 ns production MD simulation for this system has been carried out.

#### 2.6 Analysis

Before performing any analysis, we first wrapped the trajectory to account for PBC, as in Exercise 1 for the PS system. The gmx command remains the same:
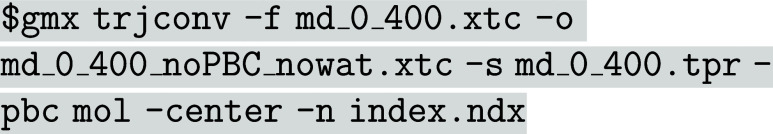
Here, we selected the POPC group to be centered in the simulation box and chose POPC_SOD_CLA (everything except water) as the output group. The water-free .tpr file (md_0_400_nowat.tpr) and water-free index file (md_0_400_nowat.ndx) files were generated the same way as in exercise 1 (section 1.4 Analysis). Using the resulting md_0_400_noPBC_nowat.xtc trajectory, we then calculated various membrane-related properties, discussed below.

##### 2.6.1 Membrane Thickness and Area per Lipid

Membrane thickness was defined as the average distance between the phosphorus atoms in the upper and lower leaflets of the POPC membrane (Figure 6(a)). A prewritten tcl script for calculating membrane thickness is provided in the directory Tutorials_files/2.POPC_in_ water/analysis/mem_thickness. The tcl script can be executed directly in the VMD environment through the TkConsole, or it can be run from the terminal using the command: vmd -dispdev text -e mem_ thickness.tcl from the analysis/mem_ thickness directory. The membrane thickness over the simulation time is shown in Figure 6(b). It is observed that fluctuations in the membrane thickness over the simulation time are minimal, with an average value of 39.2 Å.

**Figure 6:**
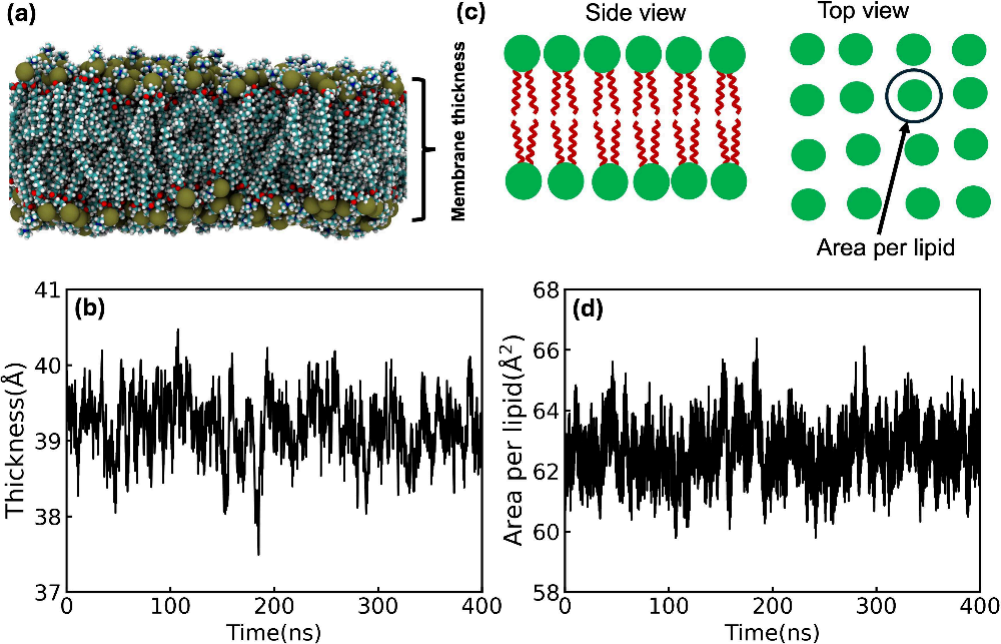
(a) Representative figure showing the thickness of the POPC membrane. (b) Thickness of the POPC membrane during the course of the simulation. (c) Schematic representation of the area occupied by individual lipids in a POPC membrane. The left panel shows a side view of the POPC structure, while the right panel shows a top-down view that highlights how the area per lipid is defined. (d) Area per lipid of the POPC membrane during the course of the simulation.

In addition, the area per lipid (*A*_lipid_) is an important parameter that describes the average surface area occupied by a single lipid molecule in the membrane plane (Figure 6(c)). It was calculated using the following equation:

2 $$A_{\mathrm{lipid}}=\frac{L_{x}\times L_{y}}{N_{\mathrm{leaflet}}}$$

where *L*_*x*_ and *L*_*y*_ are the box lengths along the *x* and *y* directions, respectively, and *N*_leaflet_ is the number of lipid molecules in one leaflet of POPC. To calculate *A*_lipid_, we first extracted the box dimensions (*L*_*x*_ and *L*_*y*_) from the trajectory using the gmx energy module in GROMACS. This tool requires an energy file with the .edr extension, generated after the simulation. The full command is
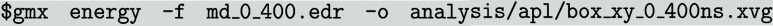
This command prompts many different options. Choose the number representing boxX and boxY, and then hit 0 to exit. The X and Y values of the box will be saved in the box_xy_0_400 ns.xvg file. Finally, the values of *L*_*x*_, *L*_*y*_, and *N*_leaflet_ are substituted at each step into the above equation to calculate *A*_lipid_ over simulation time. *A*_lipid_ shows good convergence throughout the simulations (Figure 6(d)), with an average value of 62.66 Å^2^. We used a tcl script to calculate *A*_lipid_, provided in the following directory: Tutorials_files/1.Atomistic_ simulations/2.POPC_in_water/analysis/apl.

##### 2.6.2 Lipid Order Parameter

The lipid order parameter is a measure of the structural organization of the lipid tails and influences fluidity, permeability, etc. This is calculated using the deuterium order parameter, *S*_*CD*_, which is defined as

3 $$S_{CD}=\left\langle \frac{3\;\cos^{2}\theta -1}{2}\right\rangle$$

where θ is the angle between the C–H bond vector of a lipid tail and the membrane normal (typically the *z*-axis), and the angle brackets denote a time and ensemble average. A detailed description of the methodology for calculating lipid order parameters is provided in ref 60.

We used the gmx order tool to calculate the order parameters of the POPC acyl-chain atoms. Two index files were generated, one for the sn-1 chain and one for the sn-2 chain, using gmx make_ndx. The command used to calculate the order parameters for the sn-1 chain is
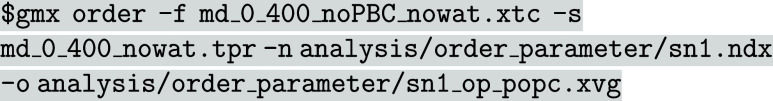


The index file sn1.ndx contains the C atoms indices of the sn-1 chain (C1–C16). This file was created using the gmx make_ndx command:
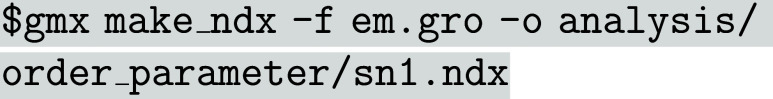
Executing this command opens an interactive window displaying all available groups in the system along with their atom counts. For illustration, the following output is obtained when running the above command:
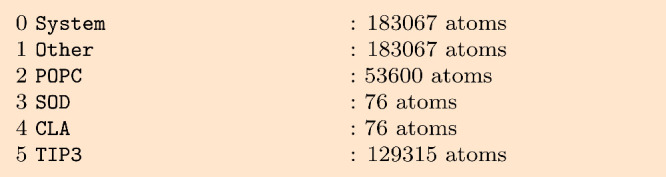


To construct the sn1.ndx, we selected the carbon atoms of the sn-1 chain using the following command:
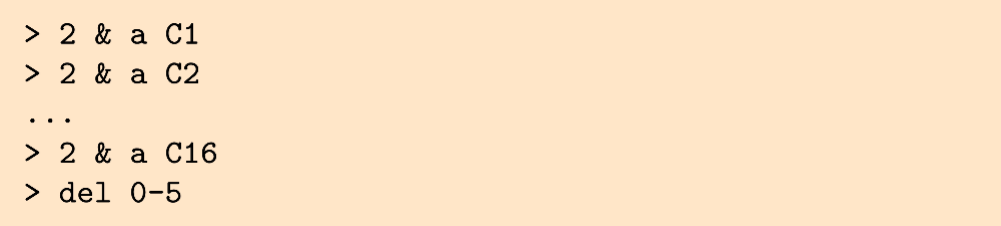
In this context, the number 2 corresponds to the POPC group, and 2 & a C1 refers to the C1 atoms belonging to that group. A similar convention applies to C2, C3, and the remaining carbon atoms of the chain. After selecting all carbon atoms belonging to the sn1 chain (C1–C16), the default groups (0–5) were deleted so that the index file (sn1.ndx) contained only the newly created sn1 group. Similarly, an index file (sn2.ndx) was created, containing the atom indices for the sn-2 chain.

Figure 7 panels (a) and (b) show the lipid order parameters of the POPC acyl chains for the sn1 and sn2 chains, respectively. The numbering of the carbon atoms is also indicated in the schematic diagram of a single POPC molecule shown in Figure 7(c). The order parameter values agree with previous studies.^60,61^ The jupyter notebook for these analyses is provided in the Tutorials_files directory.

**Figure 7:**
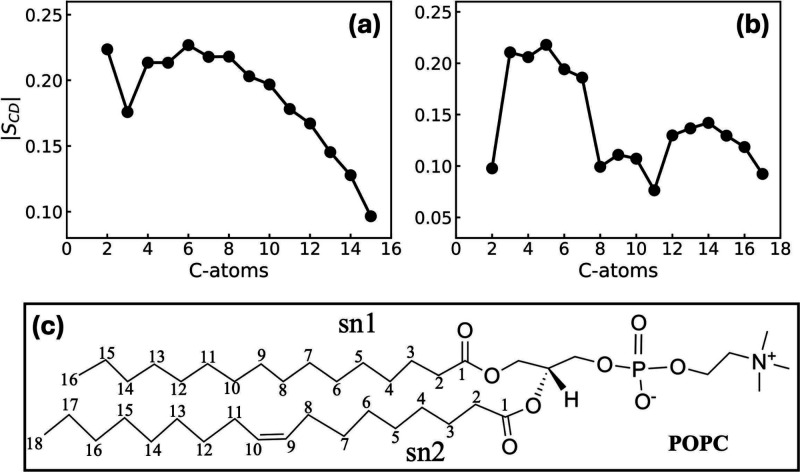
Order parameters of the carbon atoms of POPC for (a) sn1 chain and (b) sn2 chain. (c) Chemical structure of POPC showing the C atoms for which the order parameters were calculated. Note that the order parameters for the first and last carbon atoms of both acyl chains were not computed following the standard GROMACS procedure.

### Exercise 3: Perform Atomistic Simulations of Model Membrane–Nanoplastic Complex

Having established separate simulations for PS and POPC membranes in aqueous solution, we now combine these systems to investigate the interactions between PS nanoplastics and POPC membrane. To construct the POPC–PS complex, we used the equilibrated PS structure from the 400 ns PS–water simulations and inserted it into the final configuration of the 400 ns POPC–water simulations. The COM of the PS molecule was positioned above the POPC membrane surface in the upper leaflet, i.e., in the bulk water region (Figure 8). A tcl script (merge.tcl) used to build the system is provided in the Tutorials_files/1.Atomistic_simulations/3.POPC–PS directory.

**Figure 8:**
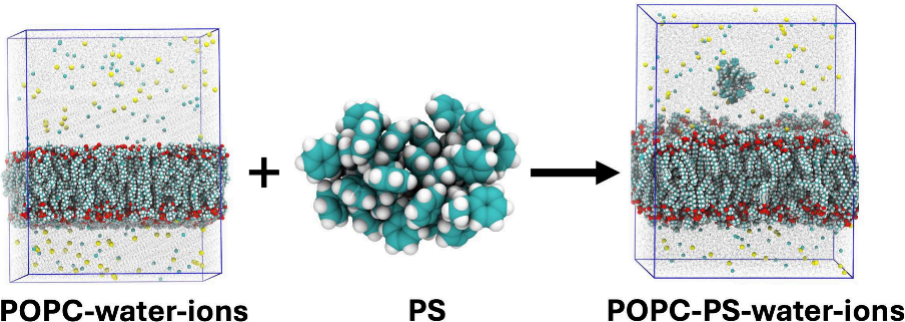
(Left) Final POPC–water–ions system from 400 ns simulations, (middle) PS extracted from the final structure of PS–water simulation, and (right) the combined structure obtained by inserting PS into the POPC–water–ions system.

The simulation workflow, including energy minimization, *NVT* equilibration, and *NPT* equilibration, is the same as in Exercise 2. Also, the GROMACS commands for gmx grompp and gmx mdrun remain identical as those in the previous exercises (Exercise 1 or 2). The energy minimization commands are shown below:

Energy minimization:
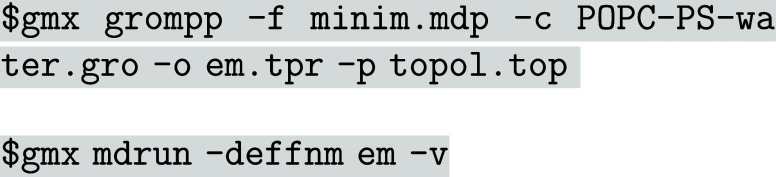


#### 3.1 Equilibrations and Production MD Simulations

The .mdp files used in this exercise are nearly identical to those used in Exercise 2, with only minor modifications in the temperature coupling section and the COM motion removal section where the tc-grps and comm-grps have been modified to POPC STYRS_STYRR SOD_CLA_TIP3, shown below.
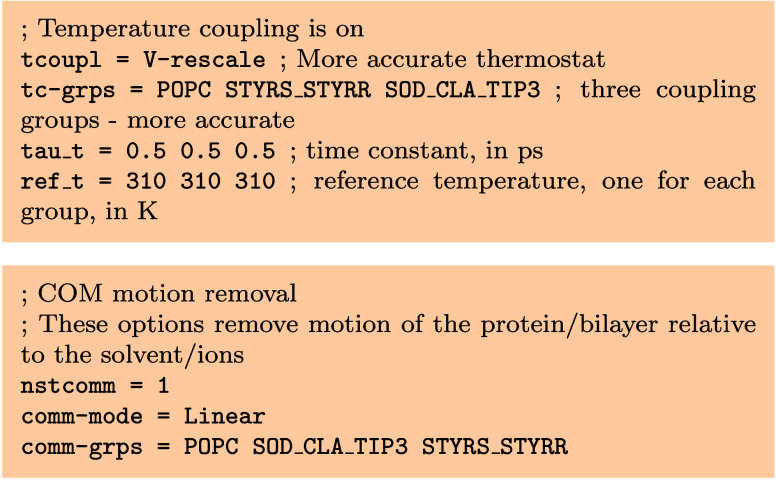


The groups STYRS_STYRR and SOD_CLA_TIP3 were created using gmx make_ndx tool discussed in Exercises 1 and 2. Likewise, the GROMACS commands for gmx grompp and gmx mdrun remain the same as in the earlier exercises:

*NVT equilibration:* 
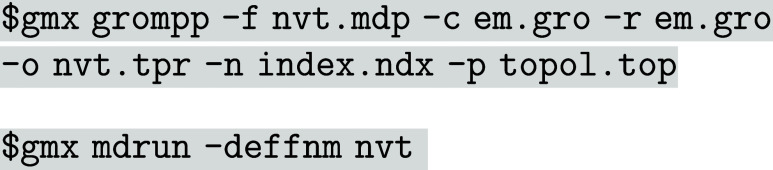


*NPT equilibration:* 
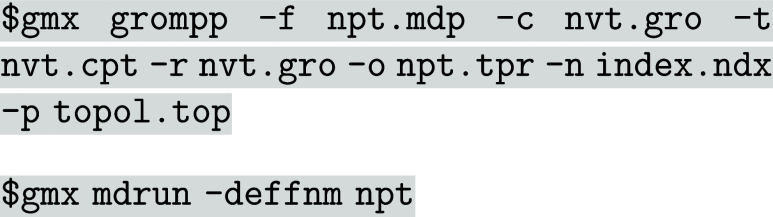


*Production MD simulations:* 
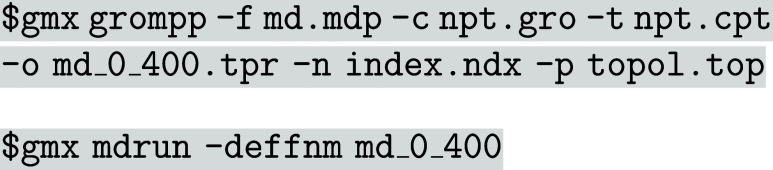
Here, a 400 ns MD simulation was carried out for the POPC–PS system. Together with the previous exercises, this exercise provides a comprehensive tutorial for exploring nanoplastic–membrane interactions with GROMACS. In the following section, we analyze properties of the membrane and PS from the 400 ns trajectory of the POPC–PS complex.

#### 3.2 Analysis

The PBC correction on the trajectory file (md_0_400.xtc) of the POPC–PS complex was performed in the same manner as in Exercise 1 or 2. Using the PBC-corrected trajectory (md_0_400_noPBC_nowat.xtc), we then calculated several structural properties of both the POPC membrane and the PS. Figure 9 panels (a) and (b) show the POPC membrane thickness and the POPC area per lipid, respectively, as functions of simulation time. Both parameters exhibit only minor fluctuations throughout the trajectory. Furthermore, their values closely match those obtained for the POPC system in the absence of PS. The radius of gyration of PS (Figure 9(c)) exhibits minimal fluctuations over time, and its values are comparable to the converged portion of the *R*_*g*_ observed in PS–water simulations (Figure 3). The lipid order parameters for the acyl chains of POPC from the POPC–PS system, along with those from the POPC–water system, are shown in Figure 10. We observe that the lipid order parameters of POPC in the POPC–water system and in the POPC–PS complex are similar. The jupyter notebook for these analyses is provided in the Tutorials_files directory.

**Figure 9:**
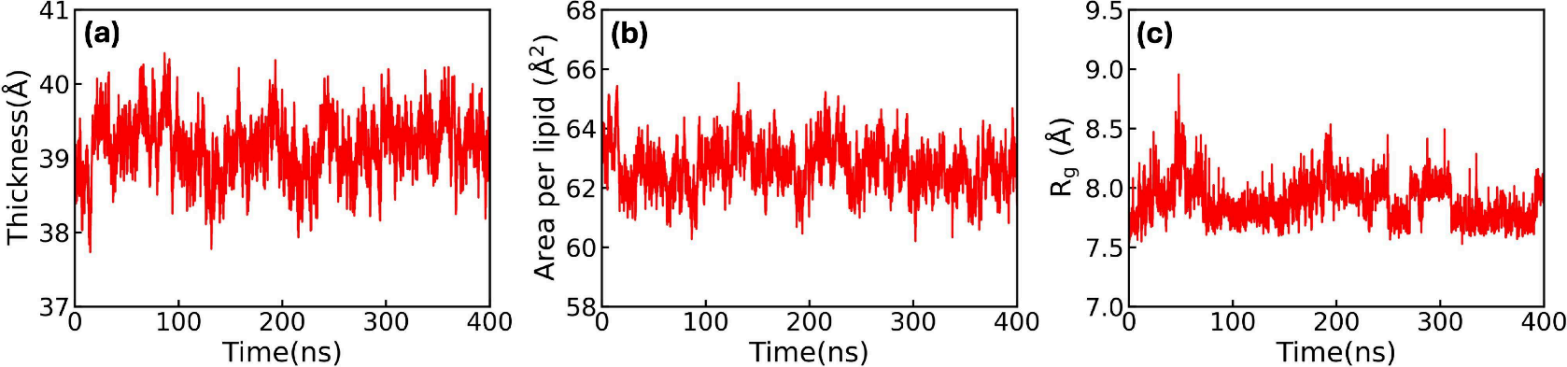
Values of (a) membrane thickness, (b) area per lipid of POPC and (c) radius of gyration of PS along the MD simulation trajectory.

**Figure 10:**
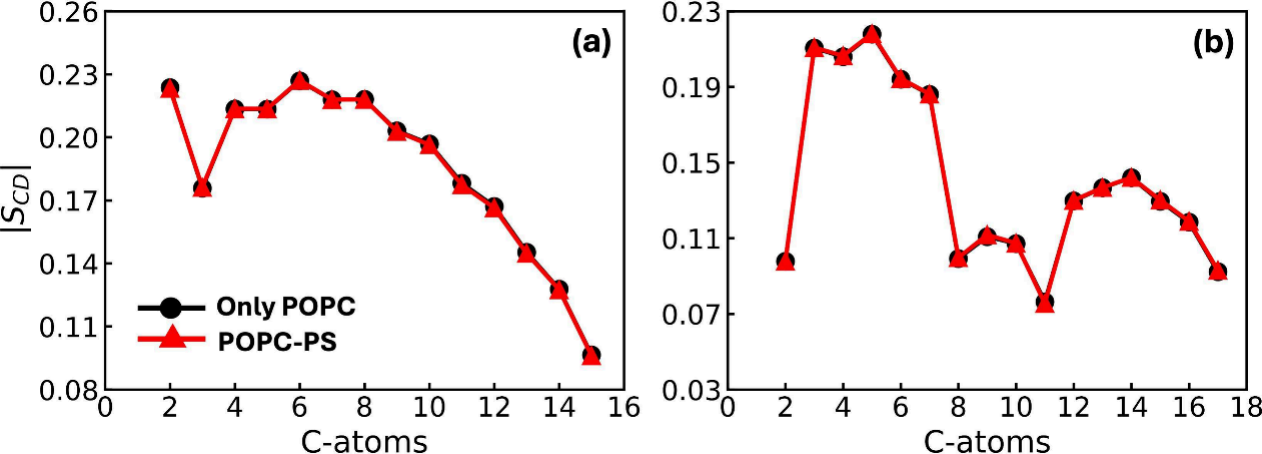
Order parameters of carbon atoms in the (left) sn1 and (right) sn2 acyl chains. The order parameters for the POPC-only system are shown in black, whereas those for the POPC–PS system are shown in red.

### Coarse-Grained MD Simulations

In previous exercises, we described the construction and simulations of the atomistic PS system in water, a POPC membrane in water, and a POPC–PS complex in water. While atomistic MD simulations provide chemically detailed insights, they are inherently limited in the system size and time scale. This restriction makes it challenging to capture processes such as large-scale membrane deformations, nanoplastic insertion, or slow diffusion. To overcome these limitations, we now turn to coarse-grained simulations, where groups of atoms are represented as single “beads”. This simplification reduces the number of particles in the system and significantly lowers computational cost, thereby enabling the study of larger systems over longer time scales.

Here, we employed Martini2 force field^8^ to construct and simulate coarse-grained models of PS chains, POPC membranes, and their complex. The systems were built using polyply and insane python tools, while the simulations were carried out using GROMACS.

### Exercise 4: Coarse-Grained MD Simulations of PS

#### 4.1 Building the System

We constructed PS chains using the Martini2 mapping scheme, in which each monomer is represented by 3 beads. To construct the coarse-grained PS system, we first generated the PS topology and coordinates using the polyply tool and solvated it using insane. Note that all the following commands in this exercise were executed from the directory Tutorials_files/2.Coarse-grained_simulations/1.PS_in_water. The procedure is as follows:

##### Step 1: Generate a PS Topology


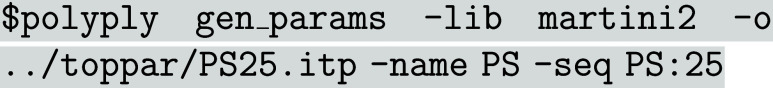
Here, the command gen_params generates the topology and parameters for PS and writes them to the PS25.itp file. Note that all .itp files are stored in the toppar directory located at Tutorials_files/2.Coarse-grained_ simulations to maintain an organized workflow. The option -name PS specifies the name of the polymer, where PS denotes polystyrene, and -seq PS:25 defines the monomer sequence length, in this case, a PS chain with 25 monomers. PS25.itp file contains charges, masses, connectivity, and the bonded parameters of PS.

At this stage, we have manually created the topol.top file, which incorporates martini_ v2.0_PEO_PS_CNP.itp file containing charges, masses, and nonbonded parameters of the molecules, including PS and water. This file can be downloaded from the Martini web server under the PS section.^62^ The topol.top file appears as follows:
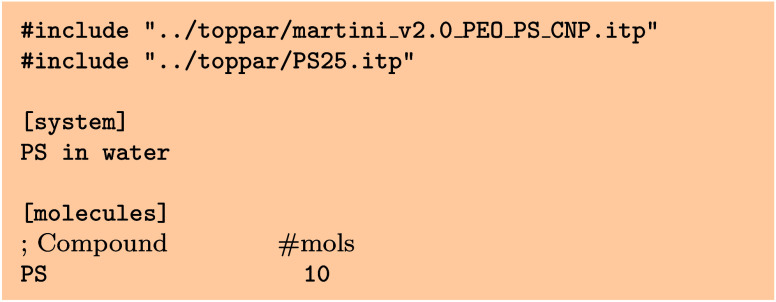


In the [molecules] section of the topol.top file, the number following PS indicates the number of polymer chains in the system. Here, we included 10 chains, but this number can be set to any desired value.

##### Step 2: Generate Initial Coordinates and Box


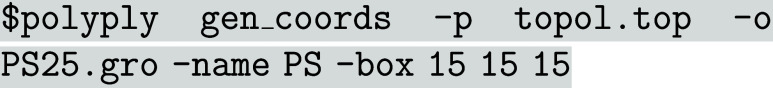
The gen_coords command generates a PS25.gro file based on the molecules specified in the topol.top file, including the number of molecules defined for PS in the [molecules] section. Here, the PS25.gro file contains 10 chains of PS inside a cubic simulation box of dimensions 15 × 15 × 15 nm^3^.

##### Step 3: Solvate with Water and Ions


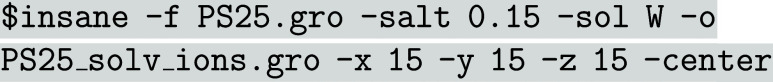
Here, we used the insane program to add Martini water beads and 0.15 M NaCl to the system. The -center option shifts the COM of the PS chains to the center of the simulation box. Since the PS chains were already randomly placed in the box in step 2, this option makes little visible difference. However, for systems containing a single molecule or a nanoparticle, using -center ensures that the molecule or nanoparticle is positioned precisely at the center of the box. A molecular diagram of PS, illustrating the mapping from atomistic to coarse-grained representation and the subsequent formation of a nanoparticle from 10 PS chains, is shown in Figure 11.

**Figure 11:**
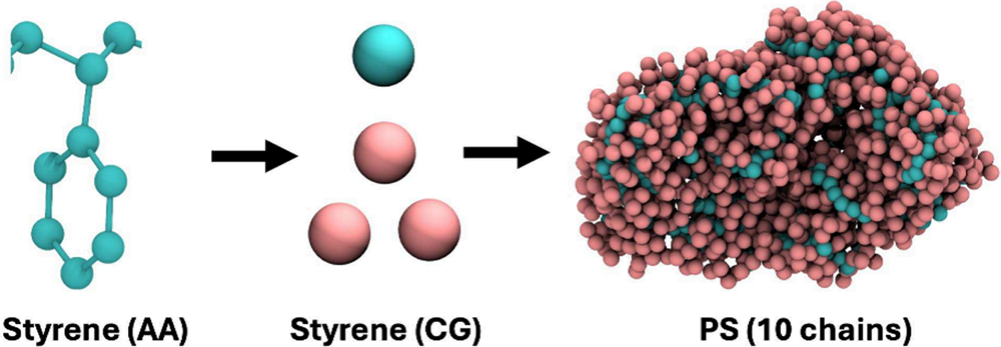
Atomistic and coarse-grained representations of PS and a coarse-grained model of a PS nanoparticle containing 10 chains, each with 25 monomers, obtained from a 400 ns equilibrium simulation. Here AA and CG refer to all-atom and coarse-grained models.

##### Step 4: Update topol.top File

After running the above command defined in Step 3, the residue name for water (W), sodium (NA *+*), and chloride (CL-) ions and their numbers are printed on the terminal. Copy those three lines and paste them at the end of the topol.top file in the [molecules] section. Note that after executing the insane command in Step 3, the numbers of water and ions printed on the terminal may vary. Therefore, readers are requested to use and appropriately modify these numbers in the topol.top file generated in their own run, particularly if the number of molecules printed on the terminal differs from those specified in the topol.top file provided in the directory Tutorials_files/2.Coarse-grained_simulations/1.PS_in_water. Now, we need to include the force field parameter files for the ions (martini_ v2.0_ions.itp), which can be downloaded from the Martini Web site.^63^ The final topol.top file is shown below:
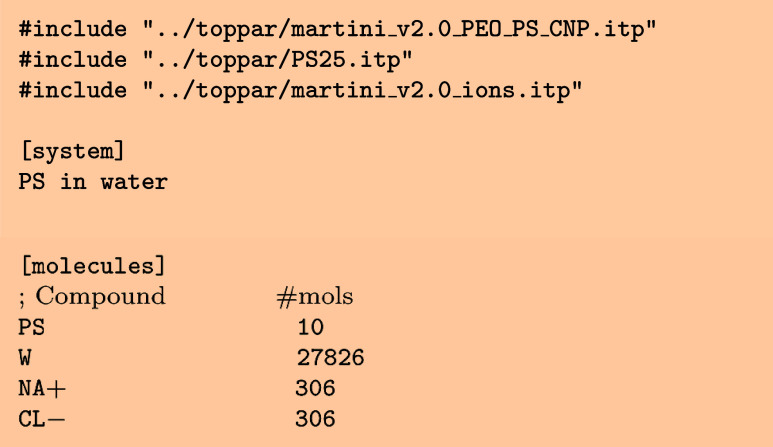


The subsequent steps, including energy minimization, *NPT* equilibration, and production simulations, follow the same procedure as described in Exercise 1 for the atomistic simulations, except that a 20 fs integration time step is used in the coarse-grained simulations. All gmx grompp and gmx mdrun commands for these steps are identical to those used in the atomistic simulations.

#### 4.2 Energy Minimization

The commands used for energy minimization are shown below:
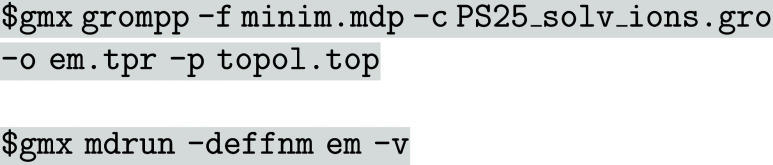


##### 4.2.1 Generating an Index File

Here, we combined the water and ions by creating an index file using gmx make_ndx, in the same way as in the atomistic simulations from Exercise 1. This index.ndx file will be used in the subsequent steps of this exercise. Note that the index.ndx file can be generated before the energy minimization step using PS25_solv_ions.gro file as well. However, since the energy minimization step does not require this file, we generate it after the minimization is complete. Running the command
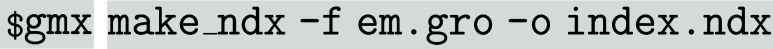
displays the default index groups and opens the interactive make_ndx interface. Within this interactive session, the groups W_ION and PS_ION were manually created using the commands 3|4 and 2|4, respectively. The W_ION group is used in the .mdp files for the equilibration and production simulations, as described in the following sections, whereas the PS_ION group can be used to generate a water-free trajectory (not performed in this exercise). The updated index file, including the newly generated groups along with the default ones, was then saved by entering the command q, shown below.
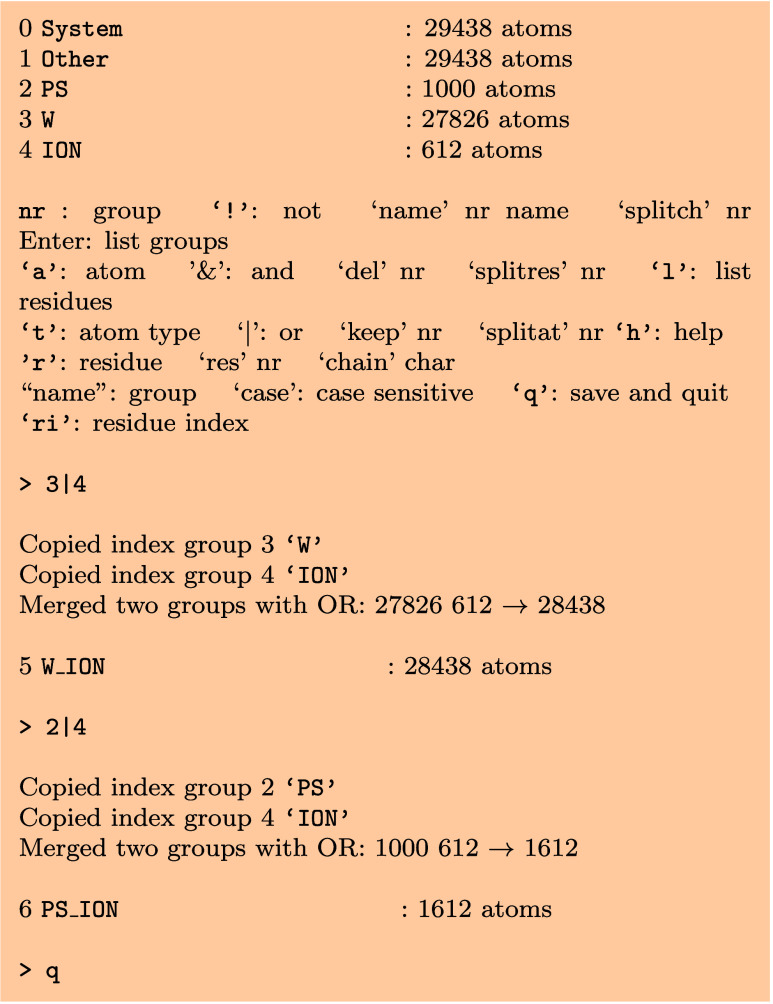


#### 4.3 *NPT* Equilibration

Note that, in this exercise, we do not perform *NVT* equilibration as it is not necessary. However, one can proceed with *NVT* equilibration before doing *NPT*. Below, we show the contents of the npt.mdp file.
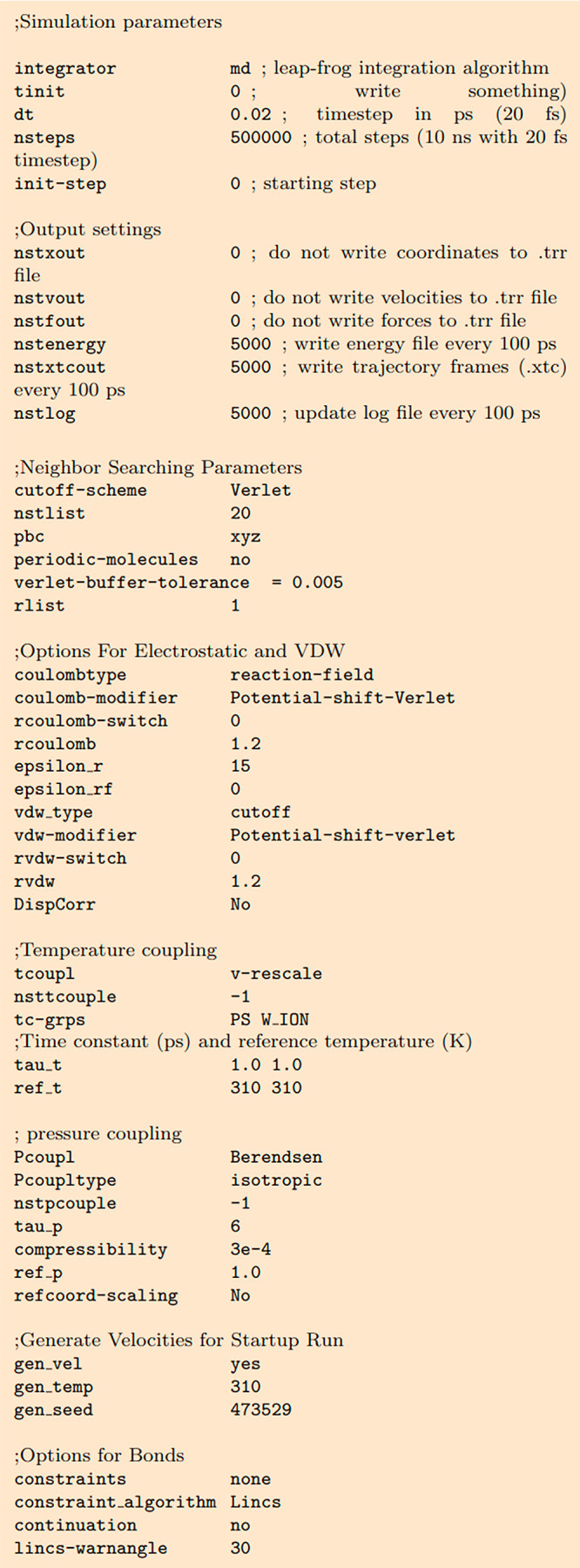


The detailed description of the .mdp parameters can be found in the GROMACS website. The commands to run the *NPT* equilibration are the following:
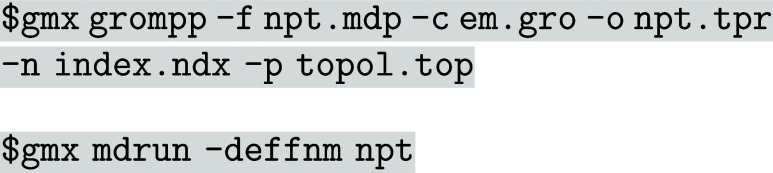
Note that no restraints were used in the npt.mdp file; however, restraints can be applied if desired, in which case the -r option must be included in the gmx grompp command. Since all simulations in this tutorial were performed using GROMACS version 2022.2, where the commands ran without issues, newer versions (e.g., 2025.4) may generate warnings regarding the Berendsen barostat. Users are advised to follow the recommendations provided in these warnings and adjust the barostat settings accordingly. If the selected thermostat/barostat settings are intentional and the user wishes to proceed despite the warning, the warning can be bypassed using the -maxwarn option (e.g., -maxwarn 1 to ignore a single warning).

#### 4.4 Production MD Simulations

The .mdp file (md.mdp) used for the production run closely follows the npt.mdp file shown above, with a few targeted modifications. The (gen_vel) keyword was removed since the production run was continued directly from the *NPT* equilibration. The pressure coupling method was changed to the Parrinello–Rahman scheme. In addition, the production simulation was extended to 400 ns, requiring the nsteps value to be set to 20000000. The commands to run this step are as follows:
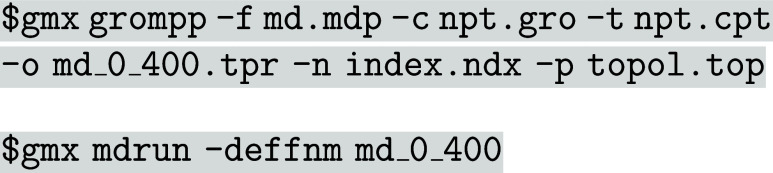


We carried out 400 ns MD simulations for this system. It is recommended to apply PBC corrections to the trajectory and to use the corrected trajectory for all subsequent analyses. The PBC correction command is the same as the one used in the atomistic simulation exercises:
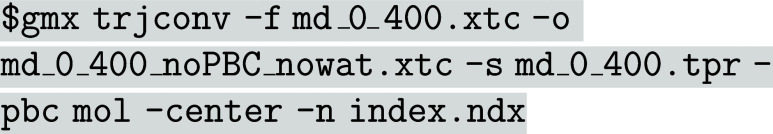
Here, we used the index.ndx file generated in Section 2.6.8. After running this command, we selected the group number corresponding to PS for centering and the group number corresponding to PS_ION for the output. This choice centers the system on PS and excludes water from the saved trajectory file, thereby reducing file size and speeding up analysis when solvent information is not required. Note that we did not perform any quantitative analysis in this exercise. However, the PBC-corrected, water-free trajectory (md_0_400_ noPBC_nowat.xtc) can be directly used to compute a range of structural and dynamical properties of PS. Examples include the radius of gyration (discussed in Exercise 1) and the end-to-end distance.

### Exercise 5: Coarse-Grained MD Simulations of POPC Membrane

In Martini2, each POPC molecule is represented by 12 beads (Figure 12). The headgroup consists of four beads: a positively charged Q0 bead for choline, a negatively charged Qa bead for phosphate, and two neutral polar beads (Na and N0) representing the glycerol backbone (Figure 3a). The two hydrocarbon tails are modeled with eight beads in total: one tail is represented by four C1 beads, and the other by three C1 beads plus one C4 bead to account for the cis double bond. Note that all commands in this exercise were run from the Tutorials_files/2.Coarse-grained_simulations/2.POPC_in_water directory.

**Figure 12:**
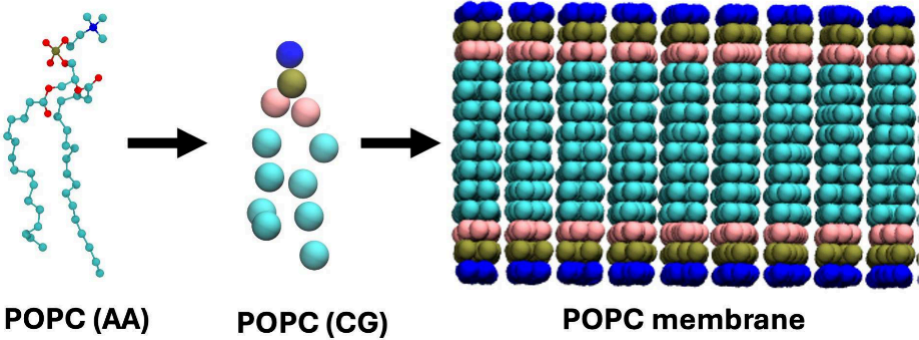
Atomistic to coarse-grained mapping of POPC. Hydrogen atoms are not shown in the atomistic structure of POPC. Here, AA and CG refer to all-atom and coarse-grained models.

We generate a POPC membrane using insane with the following command:
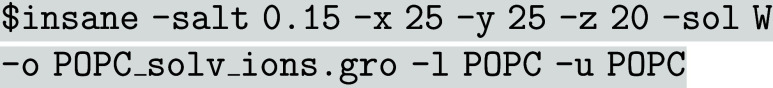
This creates a POPC membrane with 1024 lipids in each leaflet with the box dimension 25 × 25 × 20 nm^3^. The Martini water beads and a salt (NaCl) concentration of 0.15 M were added to the system.Upon execution, the command prints the molecule types and their corresponding numbers in the system to the terminal. These details should be copied and inserted into the [molecules] section of the topol.top file. The topol.top file, which is created manually, must then be updated accordingly to include the appropriate force field parameters for POPC, water, and ions, ensuring consistency with the generated system. The topol.top file is shown below:
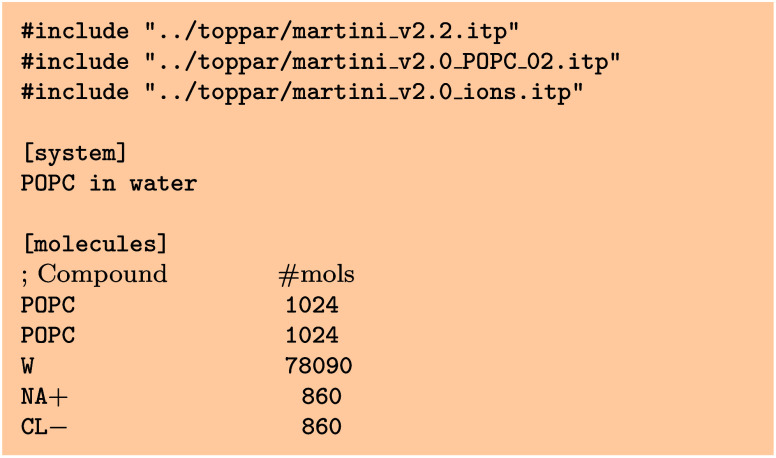


This file is provided in the Tutorials_files/2.Coarse-grained_simulations/2.POPC_in_water directory.

#### 5.1 Creating an Index File

After executing the command
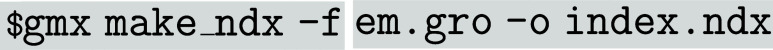
the default index groups and the interactive menu are displayed. The combined groups W_ION and POPC_ION were subsequently generated manually within the interactive make_ndx session by entering the commands 3|4 and 2|4, respectively. The updated index file, including the newly generated groups along with the default ones, was then saved by entering the command q.
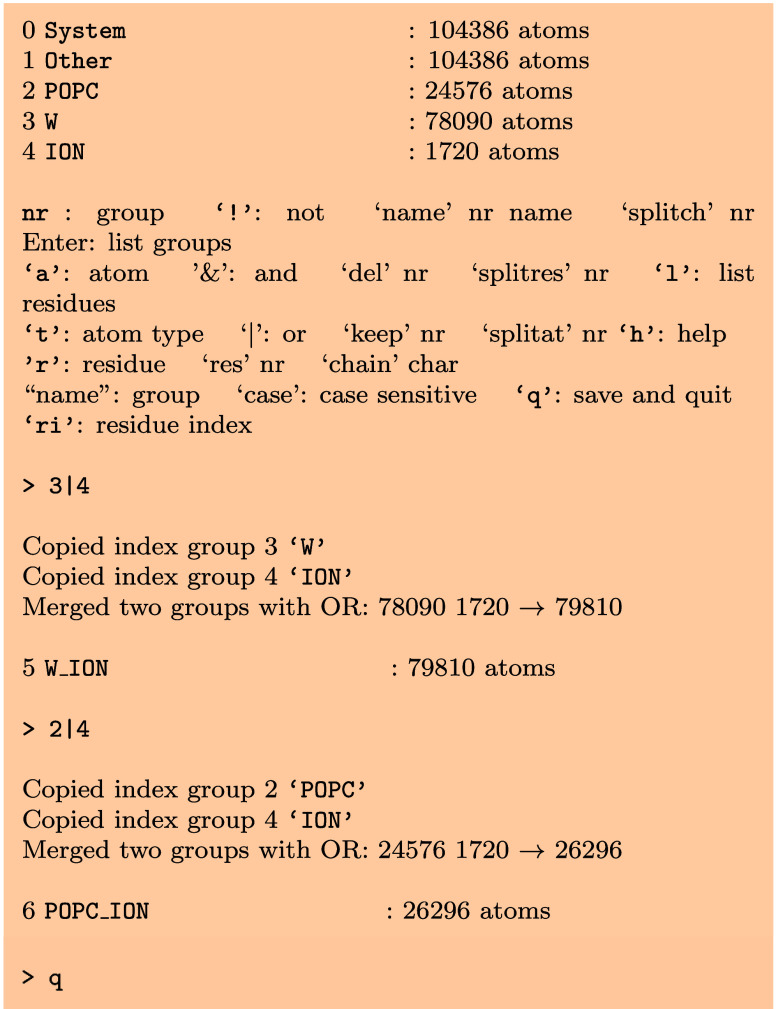


The MD simulation protocol (energy minimization, *NPT* equilibration, and production runs) was identical to that used in Exercise 4. However, unlike Exercise 4, the npt.mdp and md.mdp files used in this exercise employ a semiisotropic pcoupltype, as previously discussed in Exercises 2 and 3 for the atomistic POPC–water and POPC–PS–water simulations, respectively. In addition, the COM motion removal block was included in these .mdp files, as described in Exercises 2 and 3. The modified pressure-coupling and COM motion removal sections are shown below:
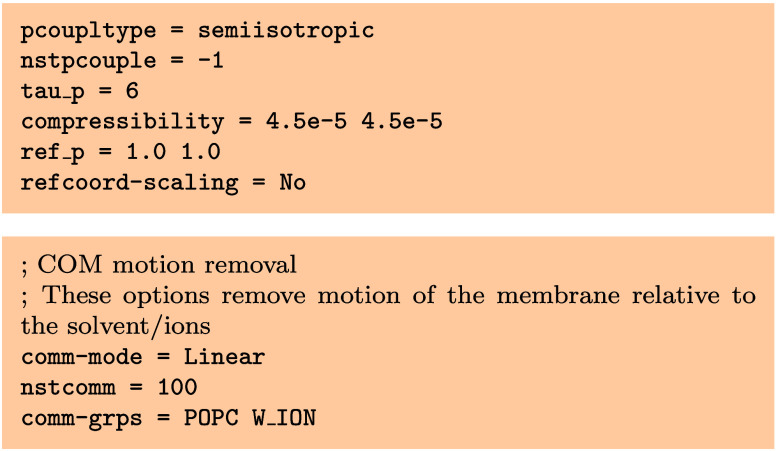


The GROMACS commands for gmx grompp and gmx mdrun remain the same as in the earlier exercises:

*Energy minimzation:* 
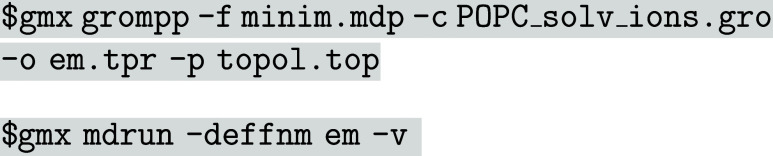


**NPT* equilibration:* 
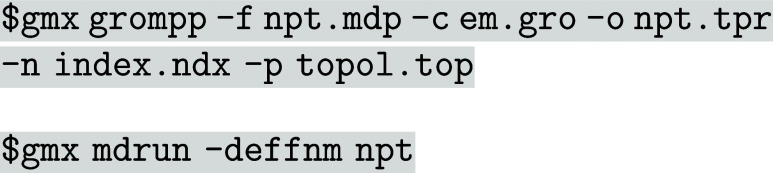


*Production MD simulations:* 
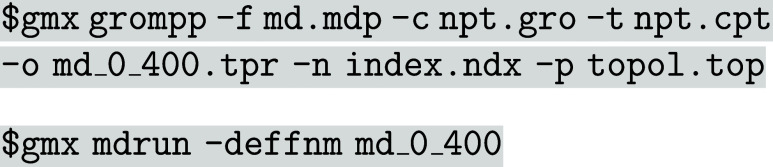


Production MD simulations were performed for 400 ns using GROMACS.

#### 5.2 Analysis

We first applied PBC corrections to the trajectory, following the same procedure as in the previous exercises, using
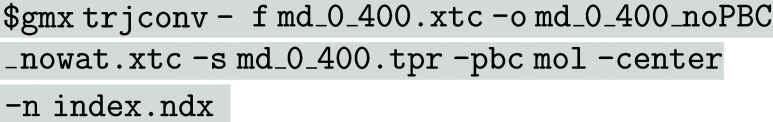


All analyses discussed below were performed using the PBC corrected water-free trajectory file (md_0_400_noPBC_ nowat.xtc).

##### 5.2.1 Membrane Thickness and Area per Lipid

The membrane thickness and area per lipid were calculated following the procedure described in the Analysis section (Section 2.6.13) of the atomistic simulations. Here, we report only time-averaged values, computed as the sum over all frames divided by the total number of frames (Table 1). However, the files containing the membrane thickness and area per lipid values as a function of simulation time are provided in the directories Tutorials_files/2.Coarse-grained_ simulations/2.POPC_in_water/analysis/ mem_thickness and Tutorials_files/2.Coarse-grained _simulations/2.POPC_in_water/analysis/apl, respectively. The average membrane thickness and area per lipid are observed to be 39.12 Å and 63.51 Å^2^, respectively. These values fall within the range reported in prior studies for the POPC membrane.^64,65^

**Table 1:** Membrane Properties from POPC–Water and POPC–PS–Water Simulations

**System**	**Thickness (Å)**	**Area per lipid (Å^2^)**	**Diffusion coefficient (POPC)** (× 10^–5^ cm^2^/s)
POPC	39.12	63.51	0.0562
POPC–PS	39.30	64.11	0.0524

##### 5.2.2 Diffusion Coefficient

The diffusion coefficient of a molecule can be estimated using the Einstein relation, which is based on the time-dependent mean squared displacement (MSD) of the molecule. A schematic diagram illustrating lipid diffusion is shown in Figure 13.

**Figure 13:**
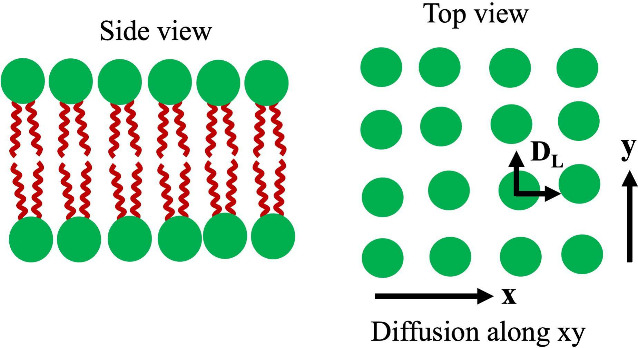
A schematic diagram illustrating lipid diffusion in the xy-plane. Left: side view of the POPC membrane. Right: top view of the same membrane, including a schematic representation of lipid diffusion. *D*_*L*_, lateral diffusion.

The Einstein relation is given by

4 $$D=\underset{t\to \infty }{\lim}\frac{\left\langle \right|r\left(t\right)-r\left(t_{0}\right){|}^{2}\rangle }{2dt}$$

where the angular brackets indicate an ensemble average over all molecules and time origins. *D* is the diffusion coefficient, **r**(*t*) is the position at time *t*, **r**(0) is the initial position, *d* is the dimensionality of the system (e.g., *d* = 1 for 1D, *d* = 2 for 2D, and *d* = 3 for 3D), and *t* is the elapsed time. Since the lipid bilayer is treated as a two-dimensional surface, we use *d* = 2 for calculating the lateral diffusion of lipids. We first calculated MSD using gmx msd tool. The diffusion coefficient was obtained from the slope of the linear region of the MSD versus time plot, using the Einstein relation (eq 4). gmx msd automatically calculates the diffusion coefficient, in addition to the MSD values over simulation time, and writes them to the output file. The MSD was calculated using the following GROMACS command:
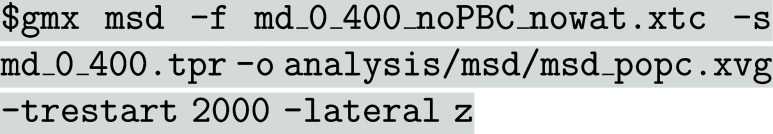
where -trestart option sets the time interval between restarts (in picoseconds) used when calculating the MSD. Here, -trestart 2000 means a new time origin *t*_0_ is started every 2000 ps (2 ns). The option -lateral z restricts the MSD calculation to lateral (2D) diffusion in the plane perpendicular to the *z*-axis. After executing the above command, the group corresponding to POPC is selected to compute the MSD of POPC lipids, from which the diffusion coefficient is subsequently obtained. The value of the lateral diffusion coefficient of POPC (Table 1) agrees with the prior studies.^66–68^

#### Finite-Size Correction on the Diffusion Coefficient Due to PBC

While the MSD approach provides a convenient and widely used estimate of diffusion coefficients, it does not inherently account for finite-size effects arising from PBC. As highlighted in prior work, diffusion is governed by long-range hydrodynamic interactions, which decay slowly (1/*r*) and give rise to non-negligible self-interactions between periodic images. As a result, diffusion coefficients obtained from MD simulations can exhibit a significant dependence on system size. For three-dimensional (3*D*) bulk systems, these finite-size effects are relatively well understood and typically lead to a systematic underestimation of the diffusion coefficient, with corrections that scale inversely with the simulation box length. Even in simple systems such as bulk water (∼2000 water molecules) in a periodically replicated cubic simulation cell, deviations on the order of 10% have been reported for commonly used system sizes.^69^ These corrections are therefore important when quantitatively comparing simulations with experiments or when transport properties are used to assess and fit interaction potentials. For a cubic simulation box, a widely used correction was derived by Yeh and Hummer,^69^ which relates the diffusion coefficient obtained under PBC, *D*_PBC_, to the diffusion coefficient of the particle in an infinite system, *D*_0_ as

5 $$D_{\mathrm{PBC}}=D_{0}-\frac{k_{B}T\xi }{6\pi \eta L}$$

where *k*_B_ *T* is the thermal energy, η is the shear viscosity, and *L* is the box length. The constant ξ ≈2.837297. This leading-order correction reflects the 1/*L* scaling of hydrodynamic finite-size effects in three-dimensional systems.

*D*_PBC_ can be computed using the gmx msd tool (eq 4), followed by the application of a finite-size correction to obtain the true diffusion coefficient *D*_0_ (eq 5). This correction accounts for the effect of PBC on the calculated diffusion coefficient. A detailed derivation can be found in the work of Yeh and Hummer.^69^

In contrast, finite-size effects are considerably more pronounced in quasi-two-dimensional (2D) systems such as lipid membranes. In these systems, hydrodynamic interactions are effectively long ranged within the membrane plane and depend strongly on the lateral box dimensions and membrane viscosity. Based on hydrodynamic theory extending the Saffman–Delbrück model,^70,71^ the diffusion coefficient under PBC in the case of *L* ≫ *L*_*z*_ can be expressed as

6 $$\begin{array}{l} D_{\mathrm{PBC}}=D_{0}-\frac{k_{B}T\xi }{6\pi \eta_{m}L}\frac{\ln\left(L^{*}-\xi \left(H^{*}\right)\right)}{1+H^{*}}\\ \xi \left(H^{*}\right)=\ln\left(1+\frac{\pi }{2}H^{*}\right)+e^{-1} \end{array}$$

where η_*m*_ is the surface viscosity of the membrane. The reduced lengths are defined as L* = *L*/*L*_SD_ and H* = *H*/*L*_SD_, where the Saffman–Delbrück length is given by *L*_SD_ = η_*m*_/η_*f*_. Here, *L*, *H*, and η_*f*_ denote the simulation box width, half the height of the water layer between two periodic images of the membrane, and the viscosity of the surrounding fluid, respectively. A detailed derivation of the finize-size corrections for the lipid membrane systems can be found in the previous literatures.^70,71^

For lipid membranes, the diffusion coefficient should be corrected using eq 6. In contrast, for polystyrene diffusing in a cubic simulation box, eq 5 is more appropriate, as originally derived by Yeh and Hummer.^69^

Note that in this study, we only calculated *D*_PBC_ of POPC lipids using the Einstein relation (eq 4). No finite-size corrections were applied to the diffusion coefficients.

### Exercise 6: Coarse-Grained MD Simulations of POPC–PS Complex

We constructed the POPC–PS complex (Figure 14(a)) by placing the final structure of PS, obtained from the PS–water simulation, above the surface of the POPC membrane in the final structure of the POPC–water simulation. This is done using a tcl script following the same procedure employed for building the atomistic POPC–PS complex described in Exercise 3 (Figure 8). The tcl script used for the construction process, merge.tcl, is provided in the Tutorials_files/2.Coarse-grained_simulations/3.POPC–PS directory. Note that all commands in this exercise were executed from this directory. Upon execution of the script (merge.tcl), a .gro file (POPC–PS_solv_ions.gro) containing POPC, PS, water molecules, and ions is generated. The numbers of water molecules, sodium ions, and chloride ions are printed in the terminal during execution of the merge.tcl script, while the numbers of PS and POPC molecules can be obtained from the topol.top files generated in Exercises 4 and 5. These values should then be updated accordingly in the topol.top file for this exercise. The topol.top file used in this Exercise is shown below:
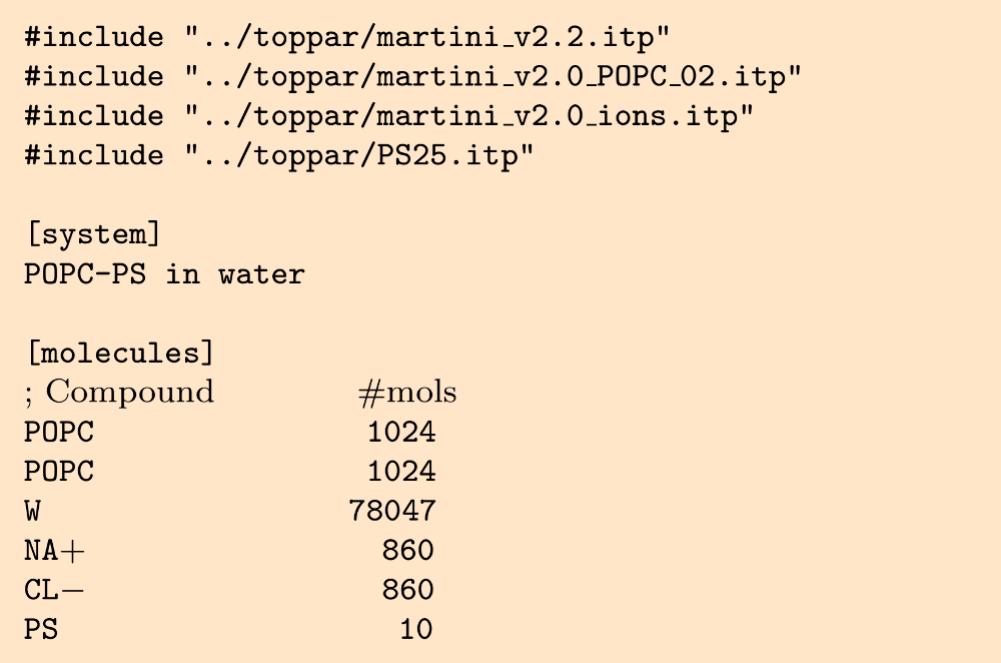


**Figure 14:**
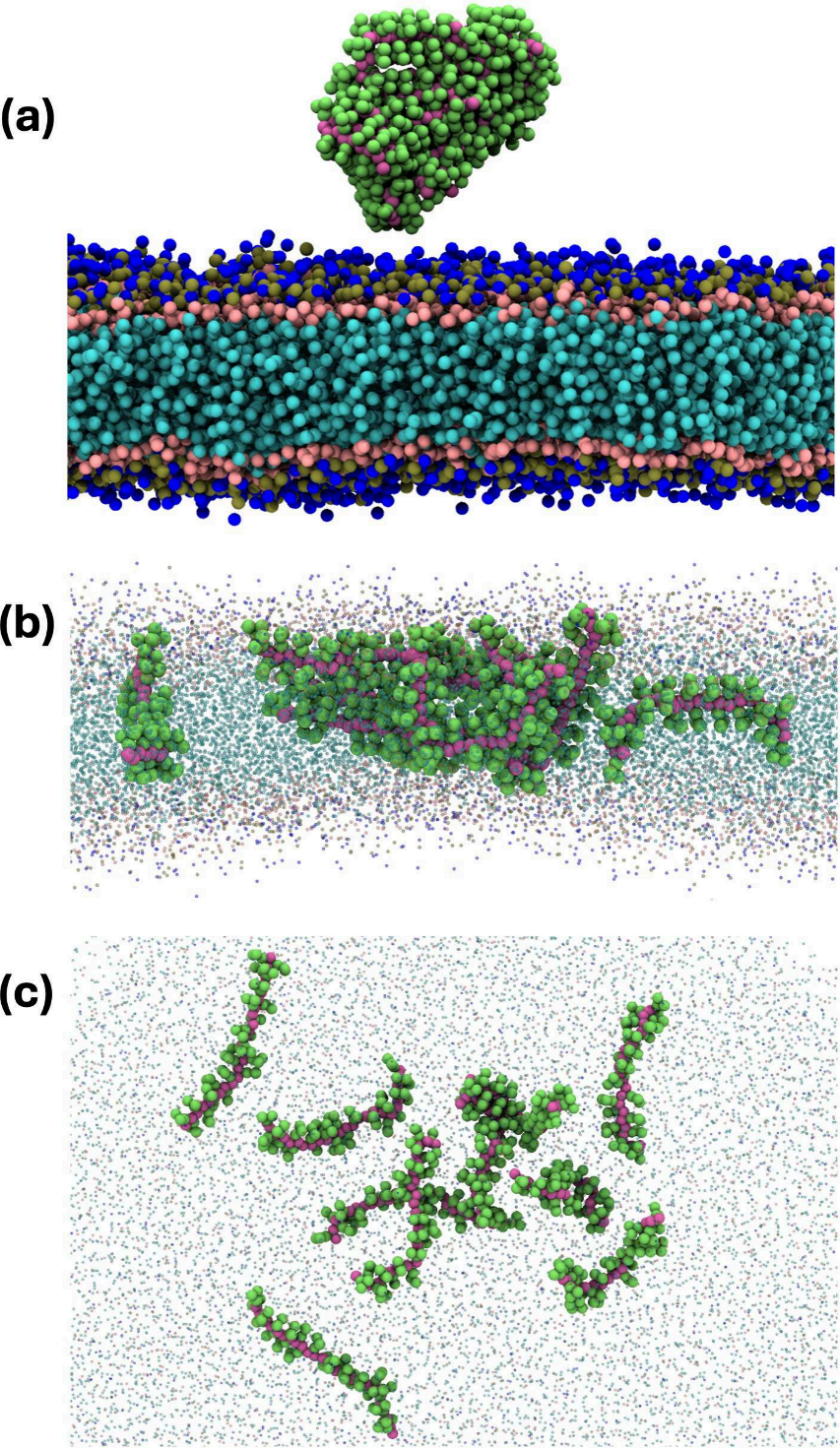
(a) Initial structure of POPC–PS complex. Both POPC and PS are illustrated in VDW representations in VMD. (b) Side view and (c) top view of the POPC–PS complex from the final structure of the 400 ns simulations. POPC is shown in CPK representation, while PS chains are shown in VDW representation. Water and ions are not shown due to clarity.

The MD simulation steps (energy minimization, *NPT* equilibration, and production run) are identical to those used in Exercise 5. The GROMACS commands for energy minimization, equilibration, and production run simulations used in this exercise are identical to those employed throughout the other exercises in this tutorial. We carried out 400 ns production simulations of the system. We observe that the PS nanoparticle penetrates the membrane, and its chains disintegrate within the membrane (Figure 14(b),(c)). In contrast, this behavior was not observed in the atomistic MD simulations described in Exercise 3, where the PS nanoparticle remained outside the membrane throughout the simulation. The reduced number of degrees of freedom in the coarse-grained model allows more efficient sampling of configurational space, which may facilitate the observation of membrane insertion events on accessible simulation time scales.

In the following, we present analyses of the POPC–PS simulation trajectory. Specifically, we calculated key membrane properties, including membrane thickness, area per lipid, and the lateral diffusion coefficient of POPC from the POPC–PS complex and compared these values with those obtained for the pure POPC membrane (Exercise 5).

#### 6.1 Analysis

We wrapped the trajectory to correct for PBC, as in the previous exercises of this paper. The gmx command used is the same:
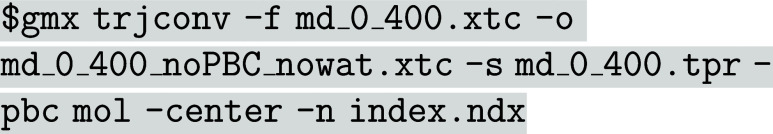


All subsequent analyses were performed on the trajectory file md_0_400_noPBC_nowat.xtc.

We report the membrane thickness and area per lipid, computed using the same procedure described in Section 2.4.7 (Exercise 2) for the atomistic simulations, in Table 1. The membrane thickness is essentially unchanged between the pure POPC membrane and the POPC–PS complex. The POPC area per lipid increases slightly in the presence of PS relative to the pure POPC system. We also computed the POPC lipid diffusion coefficient for the POPC–PS system following the same procedure described in the Analysis section of Exercise 5. The POPC diffusion coefficient shows only a minimal change between the pure POPC membrane and the POPC–PS complex (Table 1).

## Discussion and Conclusions

We introduced atomistic and coarse-grained simulations to model PS systems and POPC membranes, and their interactions, through six exercises using GROMACS. The final goal of these exercises is to investigate the interaction between PS and a POPC membrane. Atomistic simulations were first introduced to calculate the interactions at high resolution. From atomistic MD trajectories, it is evident that PS does not spontaneously insert into the membrane. As a result, none of the properties of POPC or PS changed upon interaction between POPC and PS. However, prior experimental and computational studies suggest that PS enters the membrane and becomes stabilized.^17,72,73^ It is important to note that the simulation time in this study is relatively short for this type of system, and thus, PS remains outside the membrane throughout. Consequently, the membrane and PS effectively behave as if they were in pure water. As a result, no significant changes in the structural properties of either POPC or PS are expected.

To further explore this behavior, we employed coarse-grained simulations, which allow for longer time scales and larger system sizes. Our coarse-grained simulations reveal that PS penetrates the membrane (Figure 14(b),(c)). Upon incorporation of PS into the POPC bilayer, a slight increase in area per lipid is observed relative to the pure POPC system. Notably, the change in membrane thickness is minimal, indicating that the overall bilayer structure is preserved. Moreover, the PS size in this tutorial is much smaller than that typically used in simulations to investigate membrane-nanoplastic interactions. Therefore, we expect that the PS will affect membrane properties more when a larger nanoplastic is used. Nevertheless, these modeling, simulation, and analysis strategies provide insights into the interactions of PS with lipid membranes.

The methodologies demonstrated in these exercises are broadly applicable to similar systems. For instance, the same approaches used here for POPC membranes can be readily extended to more complex lipid assemblies, whereas the procedures used for PS can be applied to various types of nanoplastics. The analyses presented in those exercises illustrate standard and essential tasks for such systems, and researchers are encouraged to explore additional analyses beyond those illustrated here. Although these exercises do not aim to generate new scientific discoveries, they provide a practical framework and guidance for researchers interested in simulating similar systems.

## Supplementary Material



## Data Availability

Sample input and output files, along with the notebook to analyze the output, are provided at doi.org/10.5281/zenodo.20533069. Note that the Tutorials_files directory contains files with the outputs that can be used in analysis. We also provide Tutorials_files2 without those so that a reader can work through the problems and run the simulations, preferably on a compute cluster.

## Notes

Additional details of modeling using CHARMM-GUI (PDF)
