# Supplementary material for: Tutorial for Atomistic and Coarse-Grained Simulations of Membrane-Nanoplastic Interactions Using GROMACS
Source: J Phys Chem B. 2026 Jul 29;130(31):7747–66. doi: 10.1021/acs.jpcb.6c01672 (PMC13455072; doi:10.1021/acs.jpcb.6c01672)
Supplement: Supplementary file 1 [file jp6c01672_si_001.pdf]

Supporting Information: Tutorial for Atomistic and Coarse-Grained Simulations of Membrane-Nanoplastic Interactions using GROMACS

Sasthi C. Mandal[†] and Atanu Acharya[*,†,‡]

†*Department of Chemistry, Syracuse University, Syracuse, NY 13244, United States*

‡*BioInspired Syracuse, Syracuse University, Syracuse, NY 13244, United States*

E-mail: achary01@syr.edu

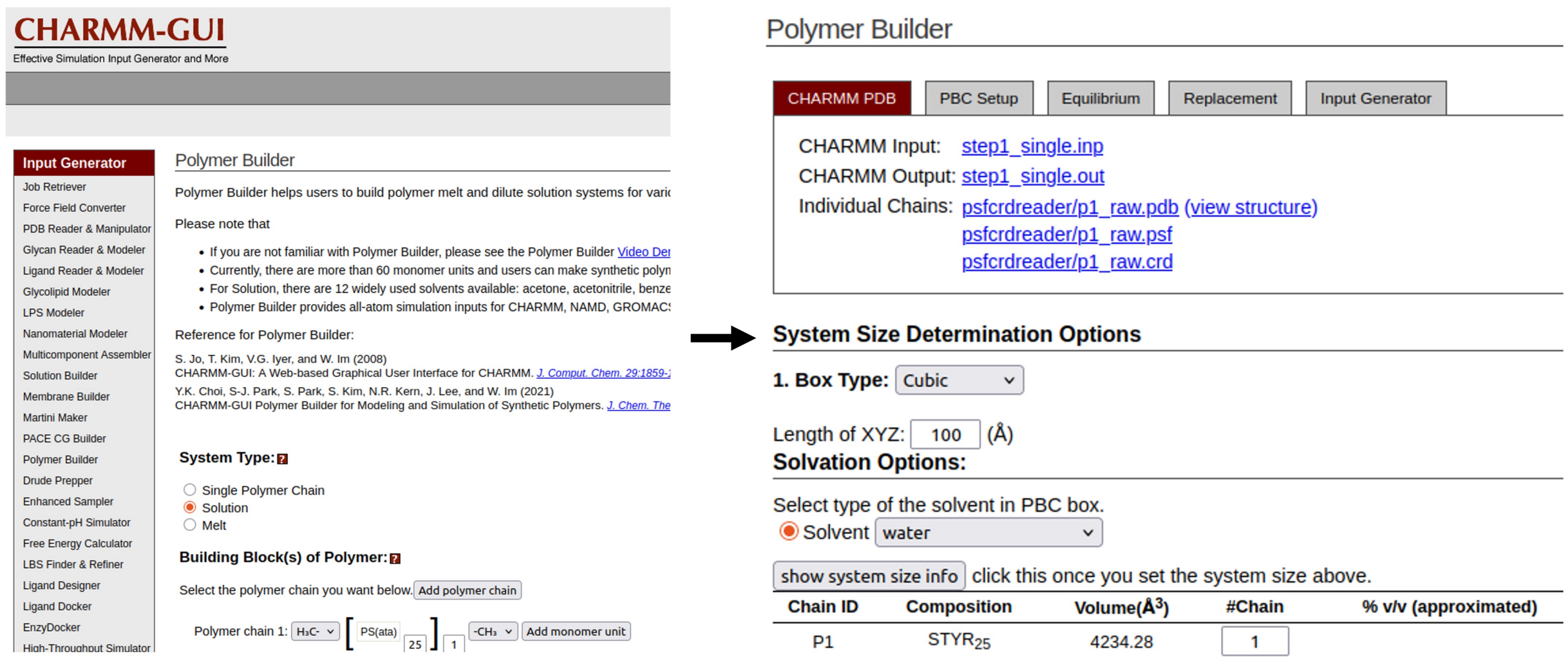

Figure S1: Representative screenshots illustrating steps 1 and 2 in the building of the polystyrene (PS) system in aqueous solution using the CHARMM-GUI Polymer Builder.

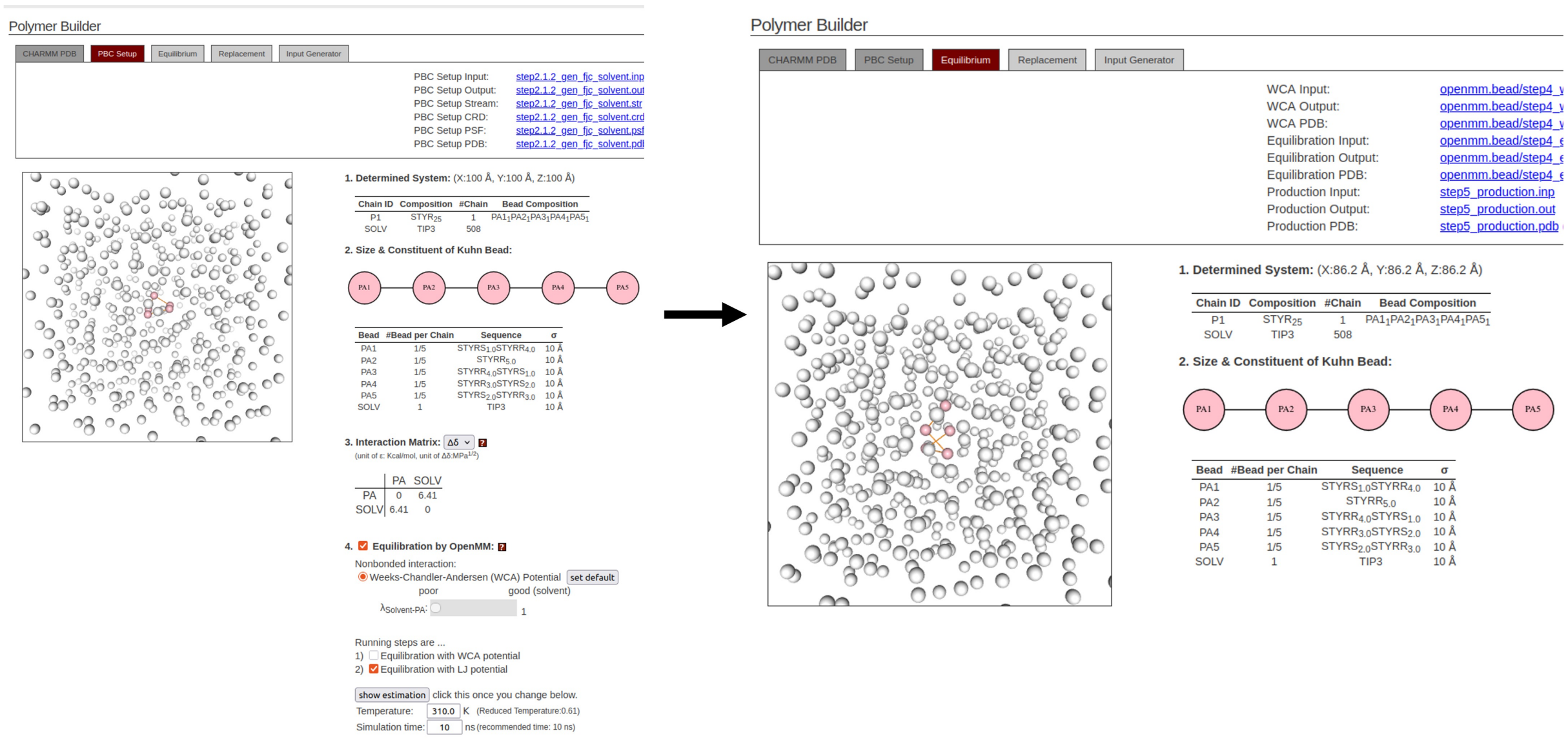

Figure S2: Representative screenshots illustrating steps 3 and 4 in the building of the PS system in aqueous solution using the CHARMM-GUI Polymer Builder.

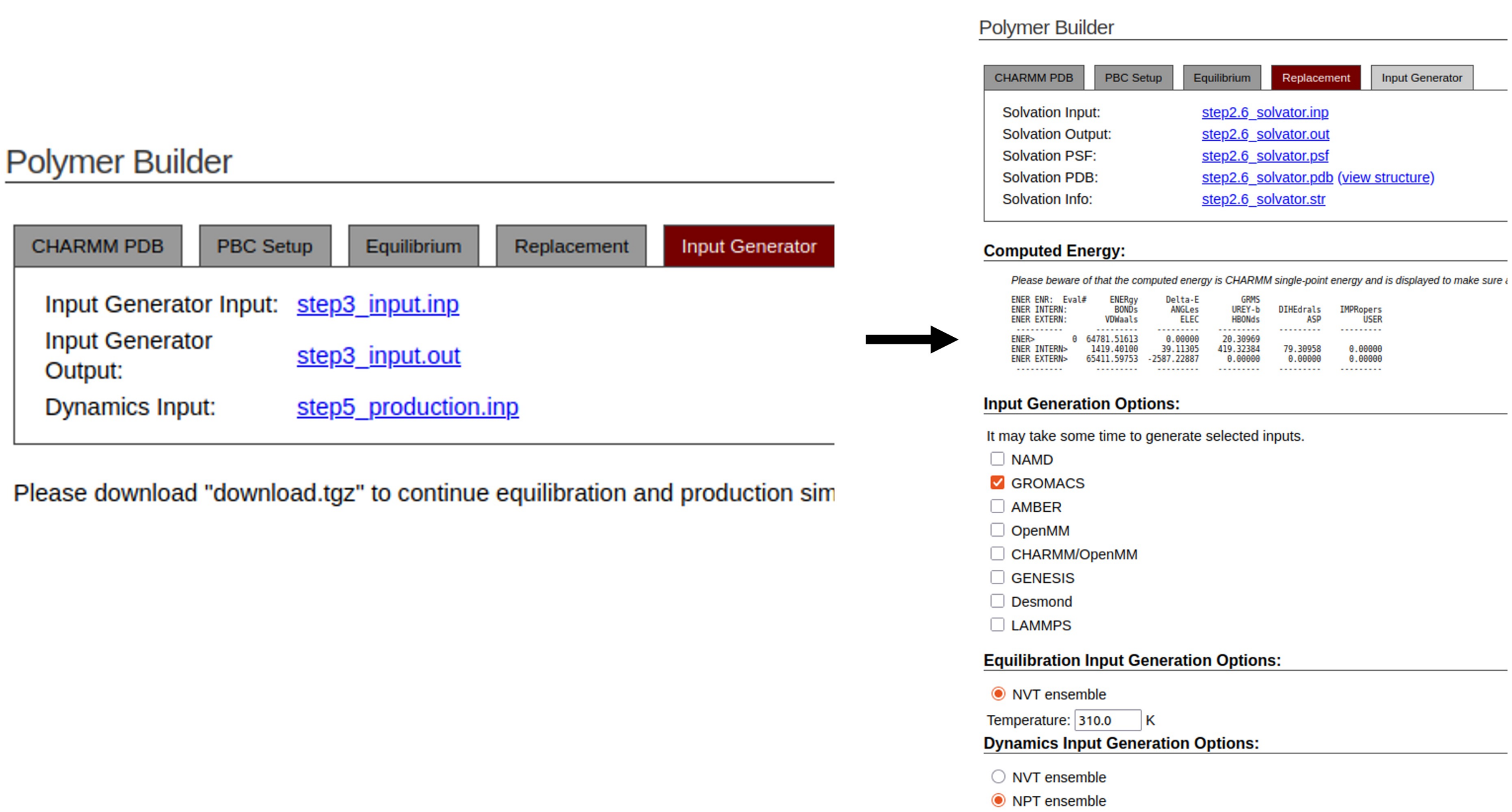

Figure S3: Representative screenshots illustrating steps 5 and 6 in the building of the PS system in aqueous solution using the CHARMM-GUI Polymer Builder.

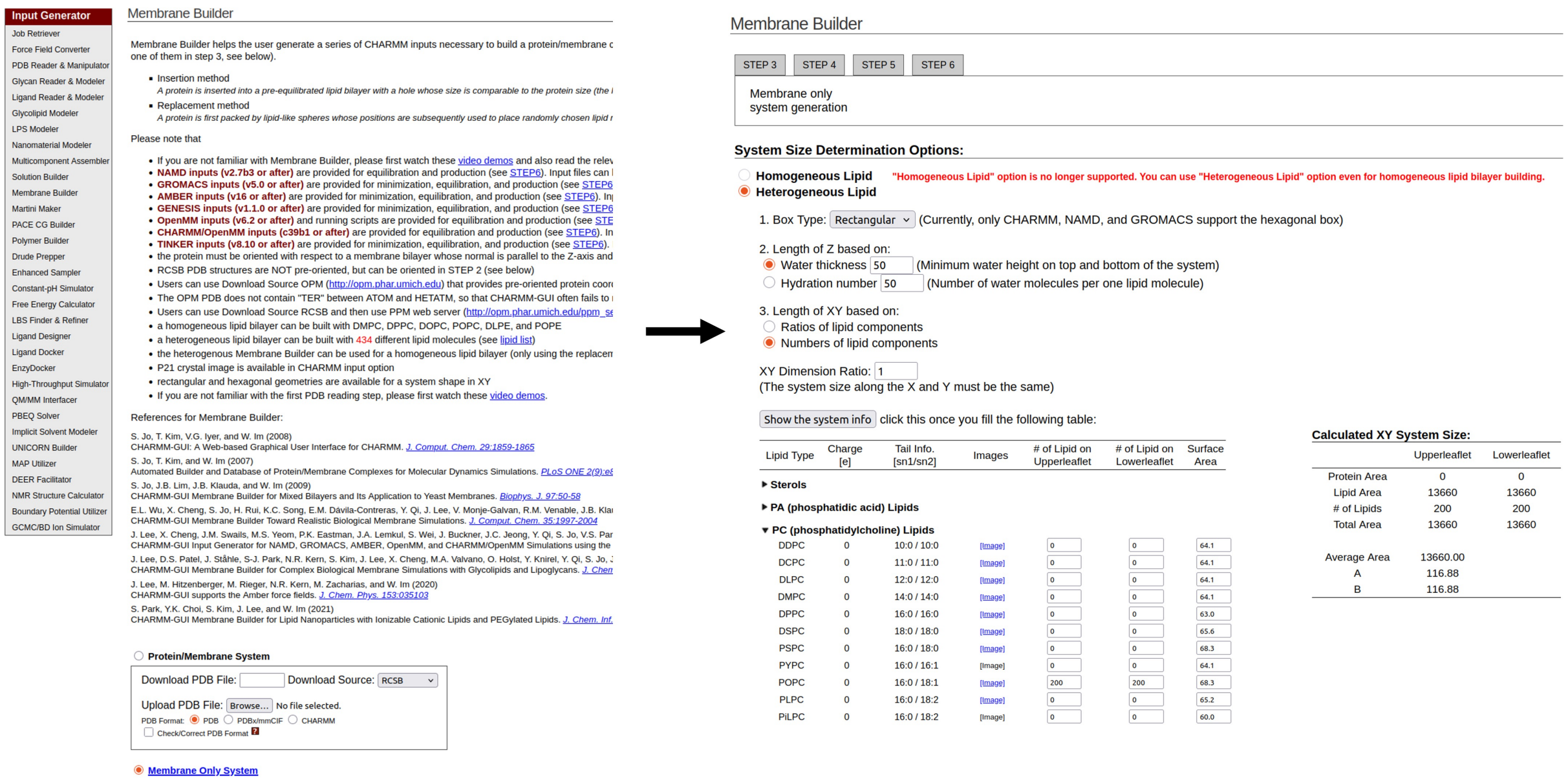

Figure S4: Representative screenshots illustrating steps 1 and 2 in the building of the POPC system in aqueous solution using the CHARMM-GUI Polymer Builder.

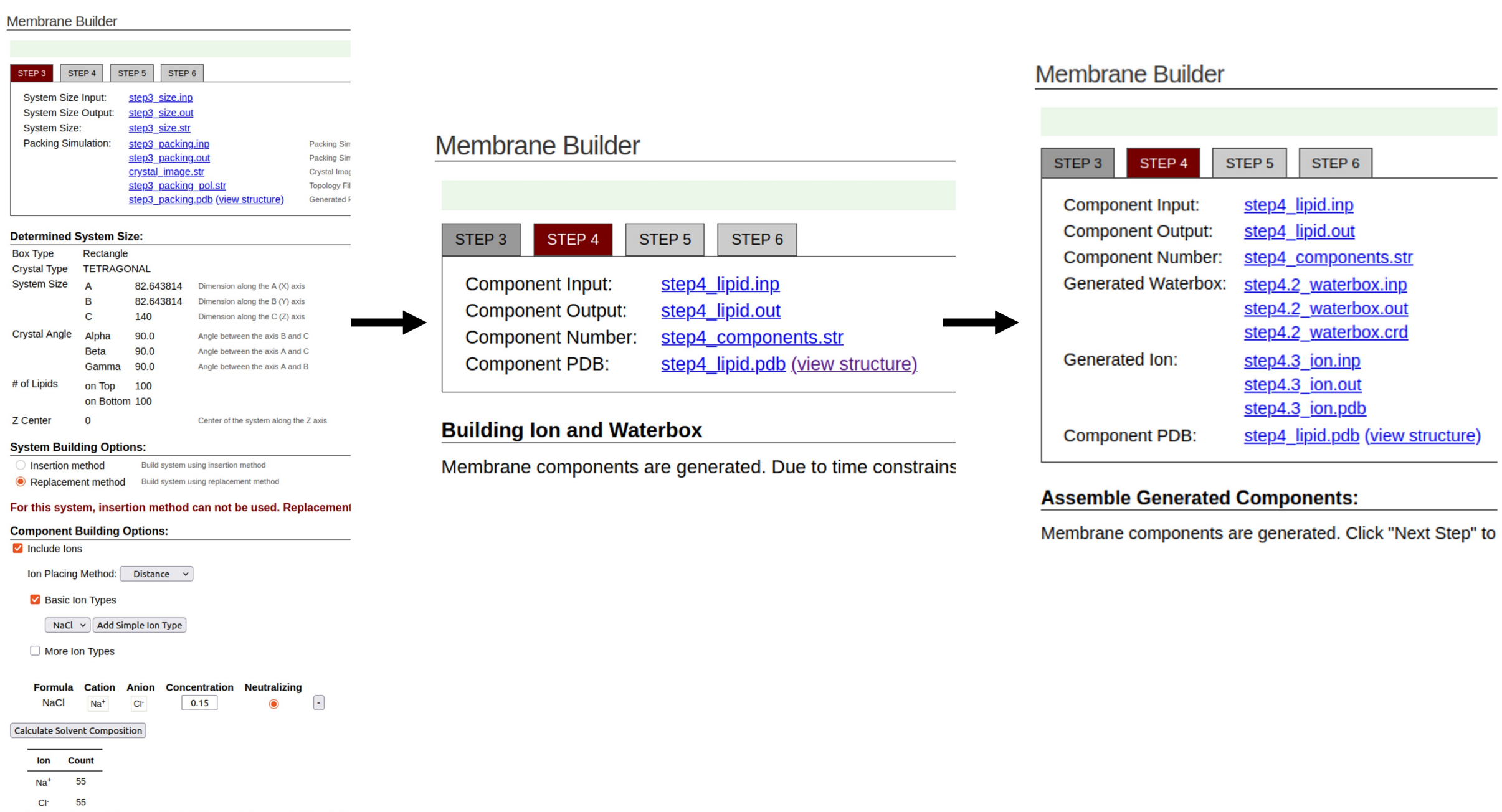

Figure S5: Representative screenshots illustrating steps 3, 4, and 5 in the building of the POPC system in aqueous solution using the CHARMM-GUI Polymer Builder.

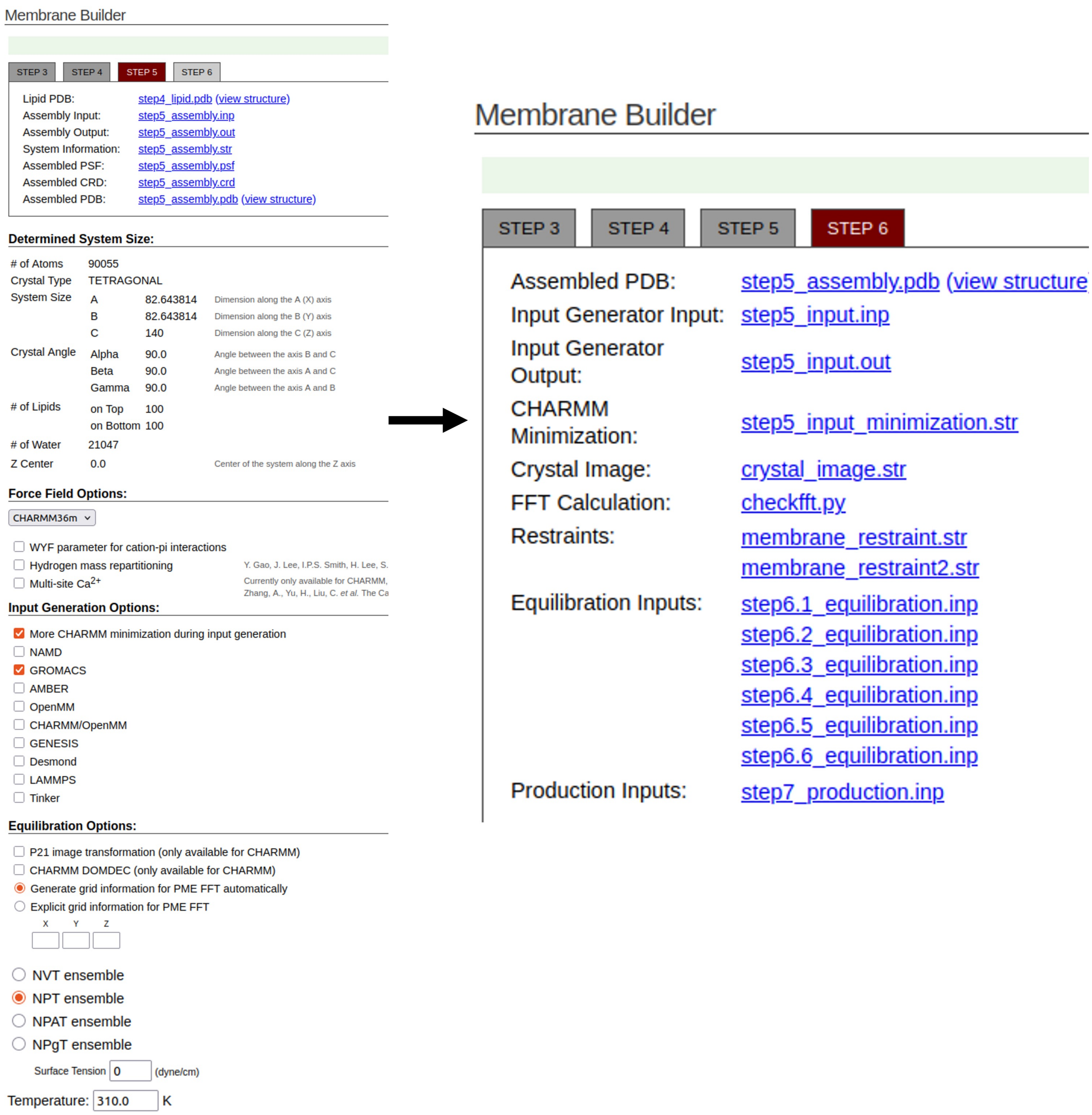

Figure S6: Representative screenshots illustrating steps 6 and 7 in the building of the POPC system in aqueous solution using the CHARMM-GUI Polymer Builder.
